# AIEgen-based theranostic system: targeted imaging of cancer cells and adjuvant amplification of antitumor efficacy of paclitaxel†
†Electronic supplementary information (ESI) available: Experimental procedures, structural characterization data and absorption spectra of compounds, as well as cell imaging results. See DOI: 10.1039/c6sc03859j



**DOI:** 10.1039/c6sc03859j

**Published:** 2016-12-02

**Authors:** Chao Chen, Zhegang Song, Xiaoyan Zheng, Zikai He, Bin Liu, Xuhui Huang, Deling Kong, Dan Ding, Ben Zhong Tang

**Affiliations:** a Key Laboratory of Bioactive Materials , Ministry of Education , State Key Laboratory of Medicinal Chemical Biology , College of Life Sciences , Nankai University , Tianjin 300071 , China . Email: dingd@nankai.edu.cn; b Department of Chemistry , Hong Kong Branch of Chinese National Engineering Research Center for Tissue Restoration and Reconstruction , Division of Biomedical Engineering , The Hong Kong University of Science & Technology (HKUST) , Clear Water Bay, Kowloon , Hong Kong , China . Email: tangbenz@ust.hk; c Department of Chemical and Biomolecular Engineering , National University of Singapore , 4 Engineering Drive 4 , Singapore 117585 . Email: cheliub@nus.edu.sg

## Abstract

Photosensitizers are generally treated as key components for photodynamic therapy. In contrast, we herein report an aggregation-induced emission luminogen (AIEgen)-based photosensitizer (TPE-Py-FFGYSA) that can serve as a non-toxic adjuvant to amplify the antitumor efficacy of paclitaxel, a well-known anticancer drug, with a synergistic effect of “0 + 1 > 1”. Besides the adjuvant function, TPE-Py-FFGYSA can selectively light up EphA2 protein clusters overexpressed in cancer cells in a fluorescence turn-on mode, by taking advantage of the specific YSA peptide (YSAYPDSVPMMS)–EphA2 protein interaction. The simple incorporation of FFG as a self-assembly-aided unit between AIEgen (TPE-Py) and YSA significantly enhances the fluorescent signal output of TPE-Py when imaging EphA2 clusters in live cancer cells. Cytotoxicity and western blot studies reveal that the reactive oxygen species (ROS) generated by TPE-Py-FFGYSA upon exposure to light do not kill cancer cells, but instead provide an intracellular oxidative environment to help paclitaxel have much better efficacy. This study thus not only extends the application scope of photosensitizers, but also offers a unique theranostic system with the combination of diagnostic imaging and adjuvant antitumor therapy.

## Abstract 2

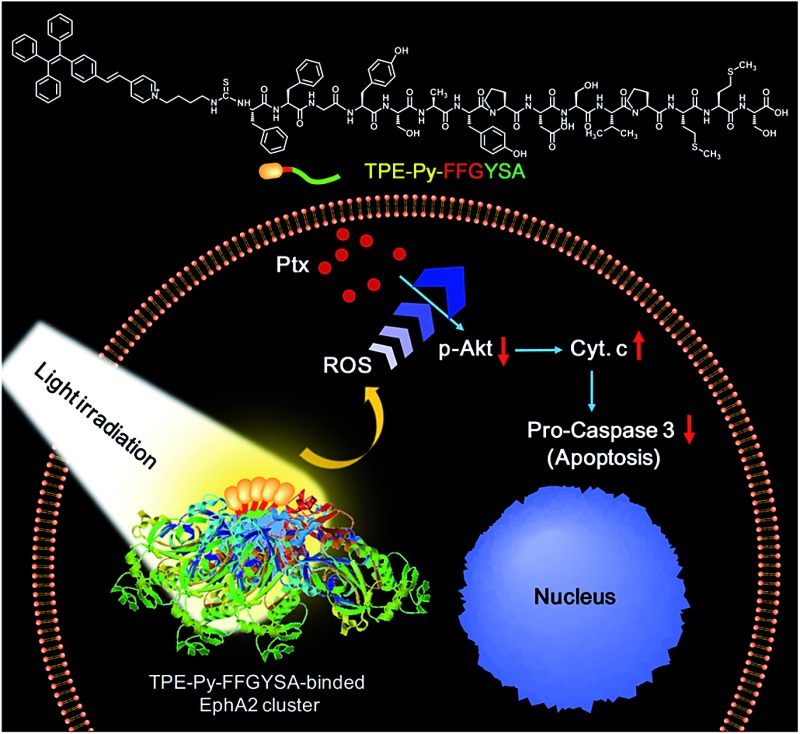
An AIEgen-based theranostic system is developed to amplify the antitumor efficacy of paclitaxel with a synergistic effect of “0 + 1 > 1”.

## Introduction

Theranostic systems that can realize diagnostic imaging and therapeutic intervention at the same time within spatial colocalization are attracting increasing research and clinical interest.^1^ Prior to treatment of many severe diseases (*e.g.*, cancer), it is necessary to conduct diagnostic imaging to visualize the focus location.^2^ Among diverse imaging techniques, fluorescence imaging is an excellent alternative due to the advantages of superb sensitivity, manoeuvrable instruments, low cost and reliable safety.^3^ Furthermore, a considerable number of fluorescence imaging agents are perfectly suitable for theranostic application, as they can undergo photophysical and photochemical processes under light irradiation to generate toxic reactive oxygen species (ROS) *in situ*.^4^ However, traditional fluorescent materials often suffer from numerous disadvantages that greatly limit their practical application in theranostics.^5^ Taking organic luminophores as an example, their working concentrations are usually in the nanomolar range to avoid aggregation-caused quenching (ACQ) effect,^6^ which leads to low photobleaching resistance and finite ROS production. This means that new luminophores are needed to overcome these limitations.

In sharp contrast to conventional luminophores, aggregation-induced emission luminogens (AIEgens) show opposite characteristics to ACQ. This endows AIEgens with the intrinsic capacity to work perfectly at high concentrations or in the aggregate state with bright fluorescence and a high photobleaching threshold.^7^ Furthermore, some AIEgen-based probes also exhibit other merits including (1) effective ROS generation in the aggregate state;^8^ (2) unique restriction of intramolecular rotation (RIR) mechanism that facilitates preparation of specific fluorescence turn-on probes;^7^ and (3) low cytotoxicity and *in vivo* toxicity allowing for safe biological applications.^9^ These unique advantages provide us with a huge amount of creative latitude to build versatile AIEgen-based theranostic systems, which will open up a new avenue for personalized treatment.

An adjuvant refers to a pharmacological or immunological agent, which functions to modify the efficacy of other agents.^10^ As adjuvants are aimed at assisting drugs or vaccines to perform better and more efficiently, they often cause negligible damage to normal tissues.^11^ Compared with the approval of a new drug that requires a time-consuming process, there may be a feasible short-cut to develop a biocompatible adjuvant that can amplify the treatment effect of FDA-approved drugs. More importantly, the development of an adjuvant provides new and greater choices for tailoring personalized treatment to individual patients.^12^ So far, reported photosensitizer-based theranostic systems (including AIEgen-based ones) have largely been designed for photodynamic therapy with the purpose of inducing the death of cancer cells, bacteria, *etc.* upon light irradiation.^8,13^ However, nearly no effort has been dedicated to study whether photosensitizers can be utilized as a non-toxic adjuvant to synergistically amplify the therapeutic effect of other chemo-drugs. Therefore, considering the unique advantages of AIEgens, we are motivated to explore a unique theranostic system based on AIEgens with the combination of diagnostic imaging and adjuvant function (AIE adjuvant), which may offer new materials and insights for the development of personalized treatment.

**Scheme 1:**
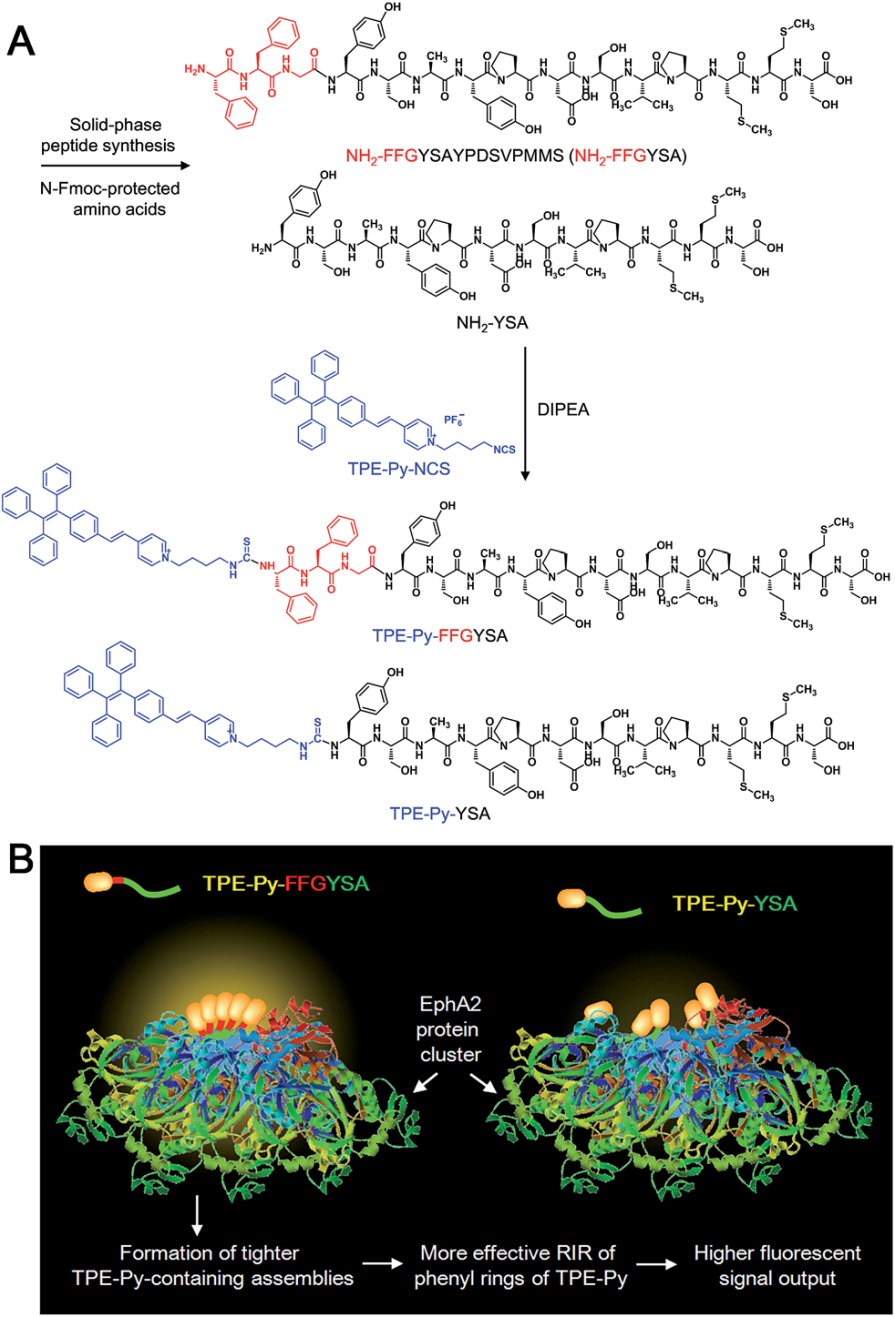
(A) Synthetic route to TPE-Py-FFGYSA and TPE-Py-YSA. (B) Schematic illustration of TPE-Py-FFGYSA and TPE-Py-YSA in imaging the EphA2 cluster.

Herein, we report for the first time an AIE adjuvant (TPE-Py-FFGYSA) that can not only specifically target and turn on its fluorescence toward EphA2 proteins, but also greatly amplify the antitumor efficacy of paclitaxel (Ptx) by acting as an adjuvant. EphA2 is a transmembrane receptor tyrosine kinase that is overexpressed in many types of cancer.^14^ In recent years, EphA2 has been accepted as one of the most attractive targets for the design of antitumor drugs.^15^ TPE-Py-FFGYSA (Scheme 1A) is composed of three parts: (1) TPE-Py as an AIEgen; (2) tripeptide FFG as a self-assembly-aided unit to promote the fluorescence output of TPE-Py *via* achieving more effective RIR, as it has been established that FF (F: phenylalanine) with an aromatic capping group usually favors supramolecular self-assembly;^16^ and (3) peptide sequence YSAYPDSVPMMS (YSA) as the targeting moiety, since YSA has been reported to selectively target EphA2.^15,17^ It is demonstrated that TPE-Py-FFGYSA is weakly emissive in aqueous solution, but shows excellent performance in visualizing EphA2 in prostate PC-3 cancer cells in a targeted and high-contrast manner. On the other hand, Ptx is well-known as one of the most widely used antitumor drugs. However, one of the hindrances of Ptx in clinical use is that several cancer cells are insensitive to Ptx.^18^ How to significantly amplify the antitumor efficacy of Ptx remains a key challenge. In this study, it is found that by optimizing the experimental conditions, EphA2-localized TPE-Py-FFGYSA can provide an intracellular oxidative environment through the light controlled generation of ROS without killing the cancer cells, which dramatically enhances the cytotoxicity of Ptx against PC-3 cancer cells, achieving the synergistic effect of “0 + 1 > 1”. To the best of our knowledge, this study represents the first example of a photosensitizer as a biocompatible adjuvant to amplify the antitumor efficacy of a chemo-drug with the effect of “0 + 1 > 1”.

## Results and discussion

An isothiocyanate-functionalized AIEgen, namely TPE-Py-NCS (Scheme 1A), was synthesized and characterized with standard spectroscopic techniques. The synthetic route toward TPE-Py-FFGYSA is shown in Scheme 1A. The peptide of NH_2_-FFGYSA was synthesized through a standard solid-phase peptide synthesis, and was then characterized using LC, ^1^H NMR, and HRMS (Fig. S1–S3†). The reaction between the isothiocyanate group on TPE-Py-NCS and the amine group of NH_2_-FFGYSA yielded TPE-Py-FFGYSA in 70% yield. The purity and chemical structure of the final product were also confirmed using LC, ^1^H NMR, and HRMS (Fig. S4–S6†). As a control, TPE-Py-YSA (Scheme 1A) without the FFG sequence was synthesized and characterized as well following the same procedures as that for TPE-Py-FFGYSA (Fig. S7–S12†).

**Fig. 1:**
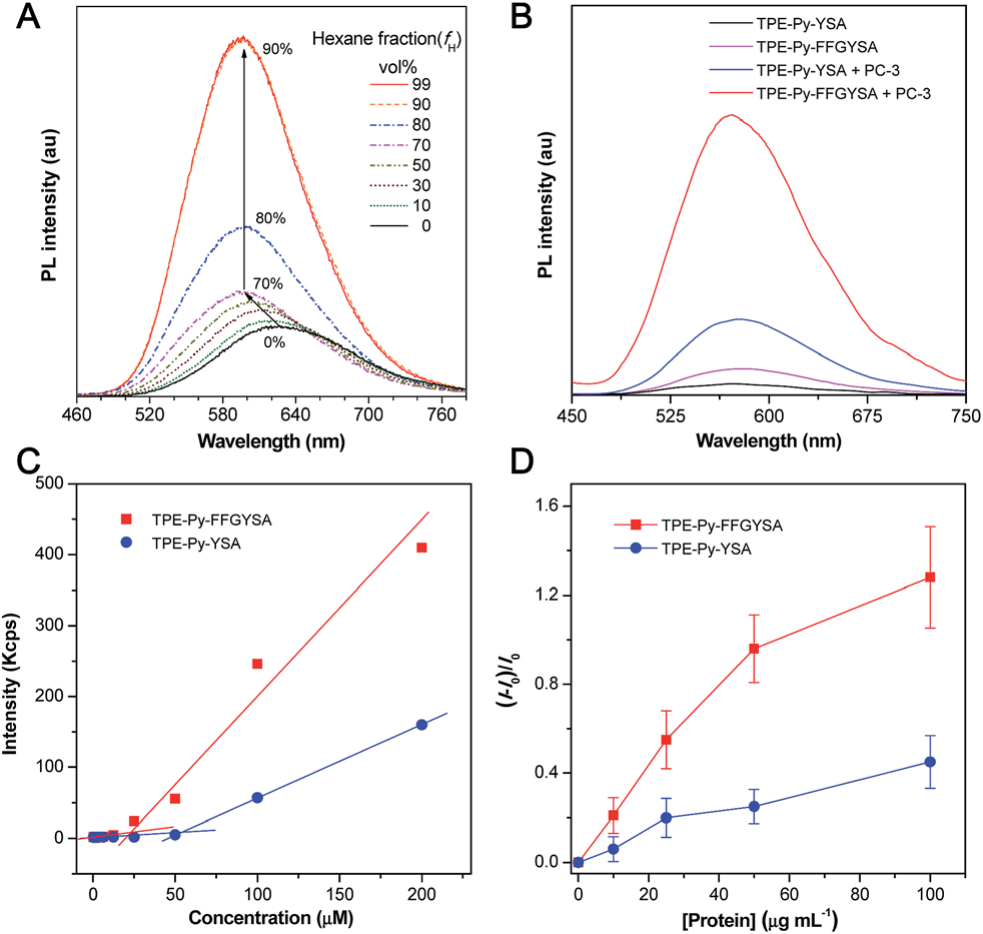
(A) PL spectra of TPE-Py-NCS (10 μM) in THF/hexane mixtures with different hexane fractions (*f*_H_). (B) PL spectra of TPE-Py-FFGYSA (1 μM) and TPE-Py-YSA (1 μM) in PBS buffer with and without the addition of PC-3 cell lysate. Excitation at 405 nm for (A and B). (C) The curves from DLS to determine CMC values of TPE-Py-FFGYSA and TPE-Py-YSA. (D) Plot of (*I* – *I*_0_)/*I*_0_ *versus* concentration of recombinant human EphA2 protein in PBS solution. *I* and *I*_0_ are the PL intensities of TPE-Py-FFGYSA (1 μM) or TPE-Py-YSA (1 μM) in the presence and absence of the protein, respectively. The data were expressed as mean ± standard deviation based on 3 measurements.

We first demonstrated the AIE characteristic of TPE-Py-NCS by measuring its photoluminescence (PL) spectra in tetrahydrofuran (THF)/hexane solvent mixtures. As shown in Fig. 1A, TPE-Py-NCS shows relatively weak emission peaking at ∼626 nm in pure THF solution. On increasing the hexane content in the THF/hexane mixture from 0 to 70%, the PL intensity is slightly enhanced with an evident blue-shift of the emission wavelength. This phenomenon should be ascribed to the typical twisted intramolecular charge transfer (TICT) effect with decreased polarity of the solvent mixture when the hexane fraction is elevated. Further increasing the hexane fraction in the mixture leads to a dramatic PL enhancement with a constant peak at ∼595 nm, which illustrates the AIE effect of TPE-Py-NCS. The absorption and emission spectra of TPE-Py-FFGYSA and TPE-Py-YSA in phosphate buffered saline (PBS) buffer are depicted in Fig. S13† and 1B, respectively. Both TPE-Py-FFGYSA and TPE-Py-YSA are weakly fluorescent in PBS buffer, although the emission of TPE-Py-FFGYSA is ∼2.2-fold higher than that of TPE-Py-YSA. It is noted that the PL spectra of TPE-Py-FFGYSA and TPE-Py-YSA are nearly unchanged when they are incubated in pure water, PBS buffer, cell culture medium with and without fetal bovine serum, respectively (Fig. S14†). This suggests that TPE-Py-FFGYSA and TPE-Py-YSA are capable of serving as fluorescence turn-on probes applicable for complex biological environments.

It has been reported that EphA2 proteins are highly overexpressed in human prostate PC-3 cancer cells,^15^ which was also confirmed by the staining experiment of PC-3 cells with commercial monoclonal anti-EphA2 antibody and subsequent fluorescent secondary antibody (Fig. S15†). Therefore, we used PC-3 cell lysates to treat with TPE-Py-FFGYSA and TPE-Py-YSA, respectively, and this was followed by PL measurements. As depicted in Fig. 1B, upon addition of PC-3 cell lysates, the emissions of both TPE-Py-FFGYSA and TPE-Py-YSA peaking at ∼575 nm are greatly enhanced. It is worth noting that, after treatment with PC-3 cell lysates, the fluorescence intensity of TPE-Py-FFGYSA is ∼3.7 times higher than that of TPE-Py-YSA, indicating the larger fluorescent signal throughput of TPE-Py-FFGYSA.

The critical micelle concentration (CMC) values of TPE-Py-FFGYSA and TPE-Py-YSA were studied using dynamic light scattering (DLS). As displayed in Fig. 1C, TPE-Py-FFGYSA has a CMC value of 24.2 μM, which implies that TPE-Py-FFGYSA molecules hardly form micelles at concentrations <24.2 μM in PBS solution. Moreover, the CMC value of TPE-Py-YSA is determined to be 53.4 μM, which is much higher than that of TPE-Py-FFGYSA. This result suggests that FFG is known as a self-assembly-aiding unit and can significantly reduce the CMC.

Titration experiments were then carried out by adding various amounts of commercial recombinant human EphA2 protein into an aqueous solution of TPE-Py-FFGYSA (1 μM) or TPE-Py-YSA (1 μM). As shown in Fig. 1D, recombinant human EphA2 results in very small changes in (*I* – *I*_0_)/*I*_0_ (∼1.3 and ∼0.5 for TPE-Py-FFGYSA and TPE-Py-YSA, respectively) even at the highest added concentration of protein (100 μg mL^–1^). According to the product specification, recombinant human EphA2 protein is highly hydrophilic, which is quite different from the endogenous ones in cancer cells that tend to form dimers and clusters.^19^ As one probe binds with one recombinant human EphA2, significant assemblies/aggregates of the probes would not form due to the good hydrophilicity of the proteins. This result also reveals that individual recombinant human EphA2 could affect the intramolecular rotations of the phenyl rings of TPE-Py to some extent, leading to slight fluorescence turn-on.

We next investigated whether the probe can specifically image EphA2 proteins that are overexpressed in cancer cells. In these experiments, PC-3 cancer cells and human smooth muscle cells were utilized as EphA2-positive and negative cells, respectively. Through antibody staining experiments, it is verified that smooth muscle cells express very few EphA2 proteins (Fig. S15†), revealing that this normal cell line can act as a good EphA2-negative control. It is important to note that most EphA2 receptors exist as dimers on the cancer cell membrane; nevertheless, after interaction with the specific ligands (*e.g.*, anti-EphA2 antibody or YSA peptide), the ligand-bound EphA2 dimers are prone to assemble into larger clusters on the membrane, followed by internalization into the cytoplasm.^19^

**Fig. 2:**
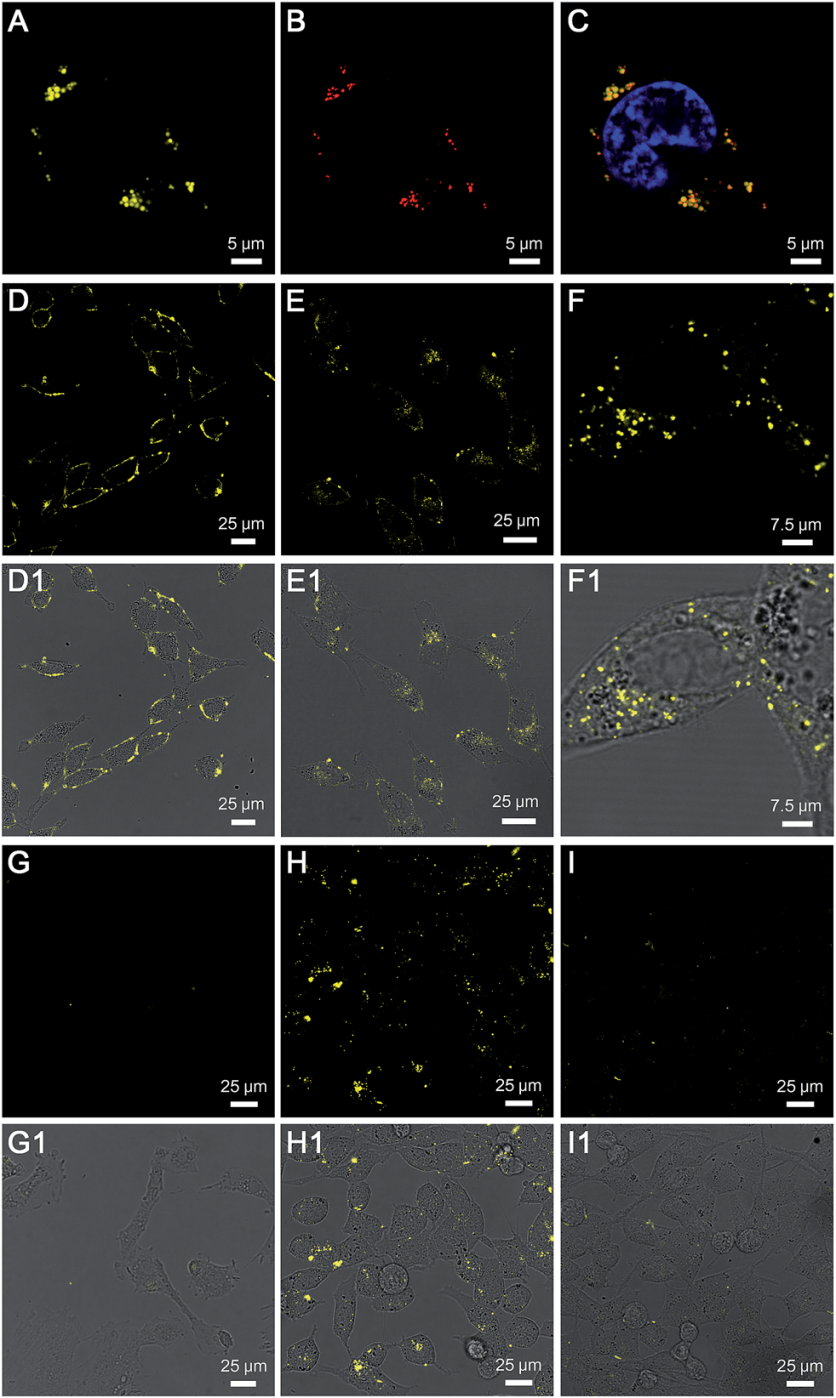
CLSM images of (A) TPE-Py-FFGYSA and (B) anti-EphA2 antibody/Alexa Fluor 633-conjugated secondary antibody co-stained PC-3 cancer cell. (C) is the overlay image of (A) and (B). CLSM images of PC-3 cells after incubation with TPE-Py-FFGYSA (D) at 0 °C for 1 h, followed by further incubation of the cells at 37 °C for (E) another 10 and (F) 60 min, respectively. CLSM images of (G) smooth muscle cells and (H) PC-3 cancer cells after incubation with TPE-Py-FFGYSA at 37 °C for 90 min. CLSM image of (I) PC-3 cancer cells after incubation with TPE-Py-YSA at 37 °C for 90 min. (D1–I1) are the corresponding fluorescence/transmission overlay images of (D–I), respectively. [TPE-Py-FFGYSA] = [TPE-Py-YSA] = 1 μM.

TPE-Py-FFGYSA (1 μM) was then used to incubate with PC-3 cancer cells. Upon incubation at 37 °C for 90 min, PC-3 cancer cells were imaged using confocal laser scanning microscopy (CLSM). As shown in Fig. 2A, distinct dots with bright yellow fluorescence are explicitly observed around the nucleus of PC-3 cells, indicating that the TPE-Py-FFGYSA fluorescence can be significantly switched on in the cancer cells. To validate that what TPE-Py-FFGYSA lit up were indeed EphA2 clusters, the PC-3 cells were also co-stained with monoclonal anti-EphA2 antibody and fluorescent secondary antibody. It was found that the yellow fluorescence from TPE-Py-FFGYSA (Fig. 2A) and the red fluorescence from the antibodies (Fig. 2B) were colocalized very well in the cell (Fig. 2C). As anti-EphA2 antibody is known to specifically bind to EphA2,^19^ the aforementioned result reasonably verifies that TPE-Py-FFGYSA is able to target and light up EphA2 clusters in PC-3 cancer cells. Additionally, the PC-3 cells were pretreated with free YSA peptides and subsequently incubated with TPE-Py-FFGYSA at 37 °C for 90 min. The CLSM image, displayed in Fig. S16†, reveals that the fluorescent signal in the PC-3 cells is significantly reduced upon blocking of EphA2 receptors. This result demonstrates that the fluorescence turn-on of TPE-Py-FFGYSA stems from its specific binding with EphA2 receptors.

To test the feasibility of TPE-Py-FFGYSA in tracking the intracellular movement of EphA2, we firstly incubated the PC-3 cancer cells with TPE-Py-FFGYSA at 0 °C, as the protein internalization is energy-dependent.^20^ After incubation at 0 °C for 1 h, intense fluorescent signals from TPE-Py-FFGYSA are observed on the membranes of PC-3 cancer cells (Fig. 2D), indicating that the EphA2 receptors are originally distributed on the cell membrane. Alternatively, after treatment with TPE-Py-FFGYSA at 0 °C for 1 h, the PC-3 cells were washed and incubated in culture medium for another 10 and 60 min, respectively, followed by imaging of the live cells with CLSM. Upon further incubation of the cells at 37 °C for 10 min, it is obvious that the yellow fluorescent signals are located in both the cell membrane and cytoplasm (Fig. 2E), suggesting that the internalization of EphA2 receptors occurs when the cells are rejuvenated at 37 °C. Dramatically, a vast majority of the fluorescent patches are observed in the cytoplasm post further cell incubation at 37 °C for 60 min (Fig. 2F and S17†), indicating the nearly complete internalization of the EphA2 clusters into the PC-3 cancer cells. This result reveals that TPE-Py-FFGYSA can monitor the intracellular movement of EphA2 in live PC-3 cancer cells.

Furthermore, the targeting capability and specific fluorescence turn-on signature of TPE-Py-FFGYSA toward EphA2 were estimated using EphA2-negative smooth muscle cells as the control. As shown in Fig. 2G, there are very few fluorescent signals detected in the smooth muscle cells upon incubation with TPE-Py-FFGYSA (1 μM) at 37 °C for 90 min, indicating that TPE-Py-FFGYSA is highly specific for lighting up EphA2 that is overexpressed in cancer cells. Moreover, TPE-Py-YSA without the FFG sequence was also utilized as a control. Fig. 2H and I show the CLSM images of PC-3 cancer cells after incubation with TPE-Py-FFGYSA (1 μM) and TPE-Py-YSA (1 μM), respectively, at 37 °C for 90 min. Compared with TPE-Py-FFGYSA-treated cells, fewer staining areas with weaker fluorescence are observed for TPE-Py-YSA-treated cells. Quantitative analysis with Image Pro Plus software suggests that the average fluorescence intensity from TPE-Py-FFGYSA-treated cells is ∼4.0-fold higher than that from TPE-Py-YSA-treated PC-3 cells, which agrees well with the cell lysate titration data (Fig. 1B). This comparative experiment shows that, compared to TPE-Py-YSA, TPE-Py-FFGYSA is capable of visualizing EphA2 proteins in cancer cells in a more sensitive and higher-contrast manner.

Recently, great research interest has focused on the “surface-induced self-assembly” strategy, which demonstrates that amphiphilic small molecules can *in situ* self-assemble into nanostructures on biosurfaces or biointerfaces even if the concentration is far lower than their CMC in bulk solutions.^21^ This phenomenon occurs because of the significant enrichment of small molecules on the biosurfaces or biointerfaces attributed to their specific interactions. Similarly, in our case, TPE-Py-FFGYSA at 1 μM cannot form assemblies/aggregates in solution, since the concentration is much lower than its CMC. However, as EphA2 receptors form clusters in cancer cells,^19^ a considerable number of TPE-Py-FFGYSA will be enriched in EphA2 clusters due to the specific binding of the protein and YSA. As a result, the significantly elevated concentration of TPE-Py-FFGYSA molecules would facilitate the formation of assemblies/aggregates of their hydrophobic moieties intra- and inter-EphA2 clusters (Scheme 1B), which vitally block the intramolecular rotations of AIEgens and open the radiative pathway, leading to great fluorescence turn-on.

The larger fluorescent signal throughput of TPE-Py-FFGYSA than TPE-Py-YSA for EphA2 imaging in PC-3 cancer cells should be attributed to the FFG sequence between the AIEgen and YSA. As illustrated in Scheme 1B, at the surface of EphA2 clusters in cancer cells, it is reasonable to envision that, compared to TPE-Py-YSA, more and tighter TPE-Py-containing assemblies/aggregates will form for TPE-Py-FFGYSA under the action of FF by virtue of its excellent self-assembly properties when capped with an aromatic group,^16^ which will thus restrict the intramolecular rotations of the phenyl rings of TPE-Py more effectively, leading to a higher fluorescent signal output. As a consequence, TPE-Py-FFGYSA can image EphA2 clusters in cancer cells in a more sensitive and higher-contrast manner, by the simple incorporation of three amino acids FFG.

**Fig. 3:**
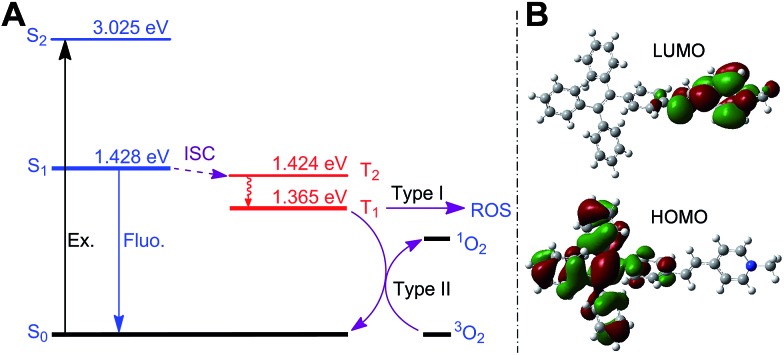
(A) Energy diagrams of TPE-Py-Me as calculated using TD-DFT and the proposed pathway of ROS generation. The energy level of ground state was set to zero. (B) Electron density distribution of the HOMO and LUMO of TPE-Py-Me based on the geometry of the excited state calculated using the B3LYP/6-31G* basis set. TPE-Py-Me is adopted as a representative of TPE-Py in the calculation model for simplicity.

**Fig. 4:**
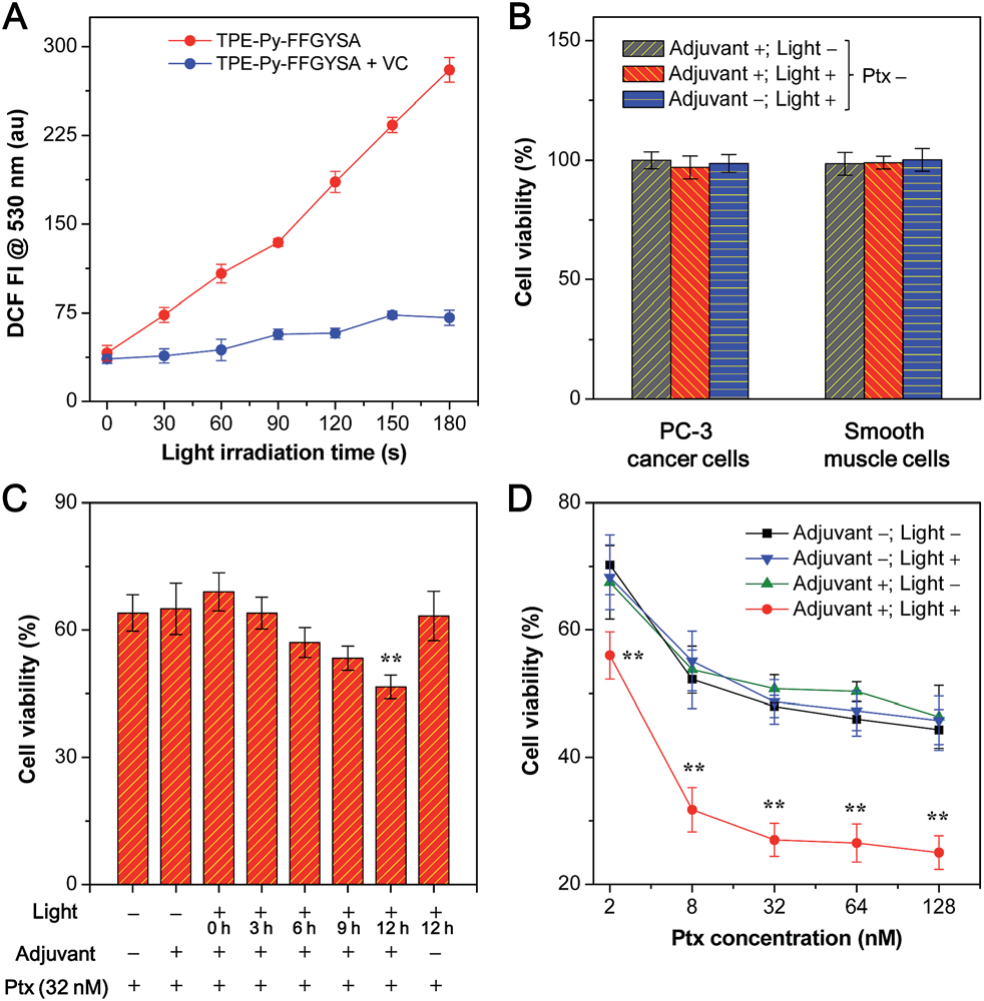
(A) Fluorescence intensity (FI) of DCF at 530 nm as a function of light irradiation time of TPE-Py-FFGYSA (1 μM) in aqueous solution with and without addition of vitamin C (VC). (B) Cell viabilities of PC-3 cancer cells and smooth muscle cells receiving different treatments of TPE-Py-FFGYSA (1 μM)/light irradiation for 48 h. (C) Cell viabilities of TPE-Py-FFGYSA (1 μM)-incubated PC-3 cancer cells after addition of 32 nM of Ptx for 24 h. Single light irradiation (0.1 W cm^–2^, 2 min) was performed at 0, 3, 6, 9, or 12 h post Ptx addition. (D) Cell viabilities of PC-3 cells after addition of various concentrations of Ptx for 48 h. PC-3 cells received different treatments of TPE-Py-FFGYSA (1 μM)/light irradiation. For (B) and (D), light irradiations (0.1 W cm^–2^, 2 min) were performed three times at 12, 24, and 36 h post addition of Ptx (Ptx is 0 nM for (B)). ** in (C) and (D) represents *P* < 0.01 *versus* the Ptx alone group (adjuvant –; light –).

Next, we studied the ability of TPE-Py-FFGYSA to generate ROS under light irradiation, which is a prerequisite to be an AIE adjuvant. Principle density functional theory (DFT) and time-dependent density functional theory (TD-DFT) investigations on a single TPE-Py molecule in both the singlet and triplet excited states were first performed. As shown in Fig. 3A, the calculated energy gap between the lowest singlet (*E*_S1_ = 1.428 eV) and involved triplet (*E*_T1_ = 1.365 eV, *E*_T2_ = 1.424 eV) excited states are considerably small (<0.1 eV), which results from a very small overlap between the highest occupied and lowest unoccupied molecular orbitals, HOMO and LUMO, respectively (Fig. 3B). The efficient single–triplet intersystem crossing (ISC) process is then facilitated. The obtained triplet excited states of TPE-Py are long-lived and reactive. They can undergo electron transfer to oxygen (type I) or/and energy transfer to a ground state triplet oxygen (type II) to produce ROS.^22^ In addition, 2′,7′-dichlorodihydrofluorescein diacetate (DCF-DA) was used as a ROS indicator, which is non-emissive but can change to fluorescent dichlorofluorescein (DCF) through a rapid oxidation reaction in the presence of ROS.^8^ As shown in Fig. 4A, upon continuous exposure of the aqueous solution of TPE-Py-FFGYSA to white light irradiation, efficient ROS production is found, as evidenced by the significant increase in the fluorescence intensity of DCF peaking at 530 nm. Such a fluorescence enhancement of the indicator could be effectively suppressed when vitamin C was added to scavenge the generated ROS. The capacity of TPE-Py-FFGYSA in ROS generation was further confirmed using another ROS indicator, 1,3-diphenylisobenzofuran (DPBF), *via* monitoring the decrease in DPBF absorbance at 418 nm (Fig. S18†).^8^ Furthermore, the ROS production of TPE-Py-FFGYSA in PC-3 cancer cells was also confirmed using DCF-DA as the indicator (Fig. S19†).

The application of TPE-Py-FFGYSA as an AIE adjuvant to enhance the cytotoxicity of Ptx was studied with MTT assay. This is done by controlling the exogenous ROS generated by TPE-Py-FFGYSA to ensure that it will not kill cancer cells, but just provides an intracellular oxidative environment to amplify the antitumor efficacy of Ptx. It is demonstrated that the 48 h viabilities of PC-3 cancer cells and smooth muscle cells after treatments with TPE-Py-FFGYSA (1 μM) without light irradiation, “TPE-Py-FFGYSA (1 μM) + light irradiation (three times at 12, 24, and 36 h; 0.1 W cm^–2^, 2 min for each irradiation)”, or pure light irradiation (three times at 12, 24, and 36 h; 0.1 W cm^–2^, 2 min for each irradiation) are all above 95% (Fig. 4B), indicating that under such experimental conditions, TPE-Py-FFGYSA is non-toxic to both cancer and normal cells even upon exposure to light. We next conducted experiments to understand when to perform the light irradiation in order to realize the synergistic antitumor effect of TPE-Py-FFGYSA and Ptx. After incubation with TPE-Py-FFGYSA at 37 °C for 90 min, PC-3 cancer cells were washed and exposed to 32 nM of Ptx. Subsequently, single irradiation with white light (0.1 W cm^–2^, 2 min) was carried out at 0, 3, 6, 9, or 12 h post addition of Ptx, which was followed by MTT assays at 24 h. As shown in Fig. 4C, upon light irradiation at 0, 3, or 6 h post Ptx addition, the PC-3 cell viabilities show no obvious difference to that without light irradiation (adjuvant +; light –). Encouragingly, when light irradiation is performed at 12 h post addition of Ptx, significantly enhanced cytotoxicity of Ptx is found. This result implies that after interaction of PC-3 cells with Ptx for 12 h, an intramolecular oxidation environment is important for the drug to give better efficacy. The possible mechanism underlying this phenomenon may be related to the cell cycle arrest of Ptx. Around 10–12 hours are needed for Ptx to arrest cells in the G2/M phase, which is the most chemosensitized phase in the cell cycle.^23^

Furthermore, after TPE-Py-FFGYSA-treated PC-3 cells were incubated with a series of doses of Ptx, light irradiation (0.1 W cm^–2^) was performed three times at 12, 24, and 36 h post Ptx addition, respectively. Each irradiation lasted for 2 min. The MTT assays at 48 h as depicted in Fig. 4D reveal that the treatments of TPE-Py-FFGYSA without light irradiation (adjuvant +; light –) and pure light irradiation without adding TPE-Py-FFGYSA (adjuvant –; light +) have negligible interference on the cytotoxicity of Ptx. It is worth noting that the antitumor efficacy of Ptx is dramatically amplified by the treatment of “TPE-Py-FFGYSA + light irradiation” (adjuvant +; light +). As calculated from the cytotoxicity curves in Fig. 4D, the IC_50_ value of Ptx alone (adjuvant –; light –) is 75.9 nM; when Ptx is combined with “TPE-Py-FFGYSA + light irradiation”, the IC_50_ value decreases to a significantly lower value of 7.8 nM, which is only 10.3% of the original IC_50_ value. A previous study has shown that amifostine as a chemosensitizer could lower the IC_50_ value to ∼14% of the value of Ptx alone, which has been well accepted as a superb performance in enhancing the antitumor efficacy of Ptx.^24^ It is also important to emphasize that “TPE-Py-FFGYSA + light irradiation” does not lead to cell death under the conditions used for the above studies (Fig. 4B). Hence, it is reasonable to conclude that with the help of light irradiation, TPE-Py-FFGYSA can serve as an effective adjuvant for synergistic antitumor therapy with Ptx to yield the effect of “0 + 1 > 1”.

**Fig. 5:**
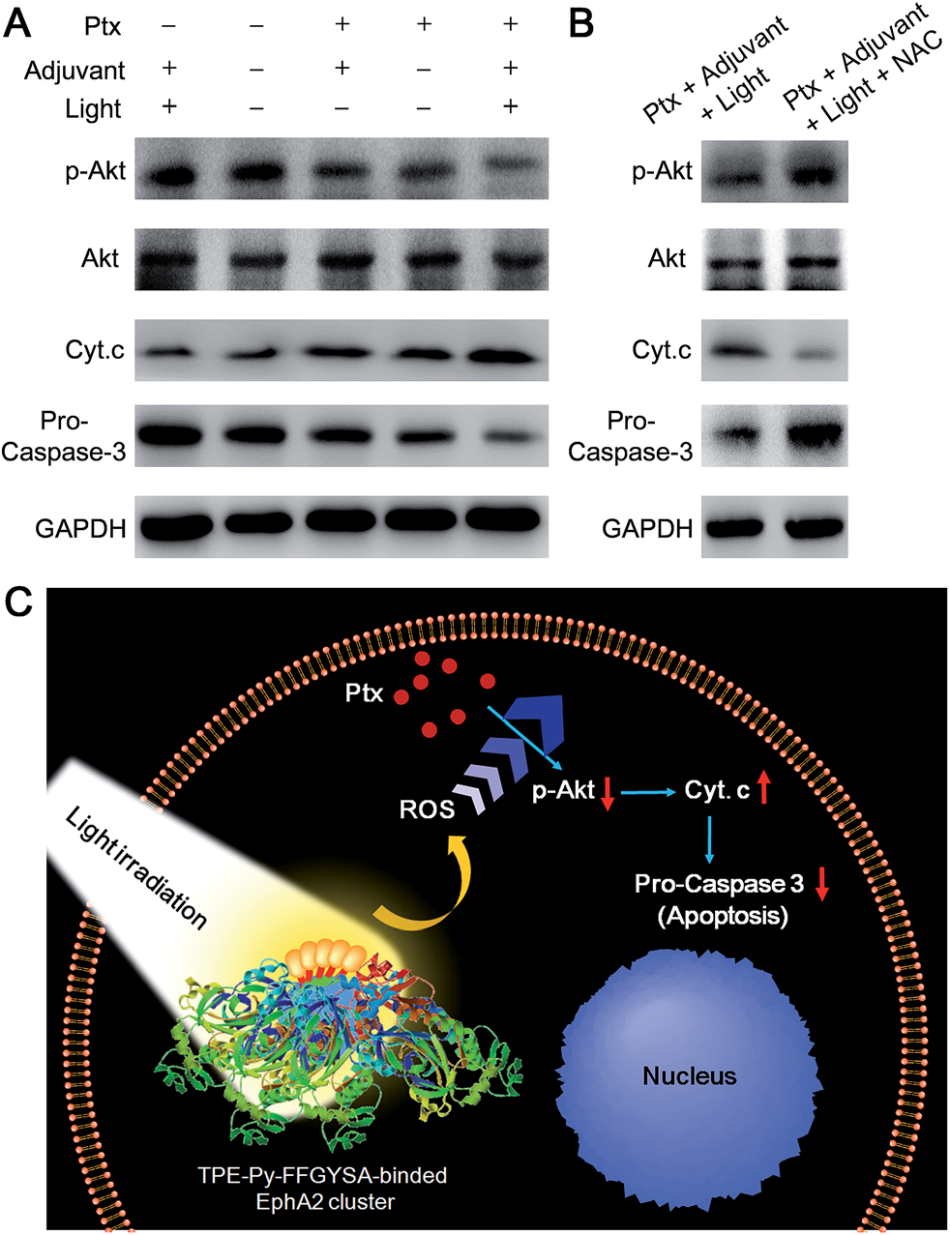
(A) Western blot analysis of various protein expressions in PC-3 cancer cells receiving different treatments. (B) Western blot analysis in the absence and presence of NAC. (C) Schematic illustration of the proposed synergistic mechanism.

We then examined the expression of related proteins using western blot to study the possible mechanism of such a synergistic antitumor effect between Ptx and “TPE-Py-FFGYSA + light irradiation”. As shown in Fig. 5A, in the absence of Ptx, “TPE-Py-FFGYSA + light irradiation” (Ptx –; adjuvant +; light +) has nearly no impact on the expression of proteins in PC-3 cancer cells compared with the untreated cells (Ptx –; adjuvant –; light –). This result verifies that “TPE-Py-FFGYSA + light irradiation” does indeed not lead to the death of PC-3 cells. Moreover, there is also no significant difference between the protein expression of PC-3 cells treated with Ptx alone (Ptx +; adjuvant –; light –) and “Ptx + TPE-Py-FFGYSA without light irradiation” (Ptx +; adjuvant +; light –), which further confirms that exposure to light is the key factor to initiate the synergistic effect. In particular, the expression of phosphylated Akt (p-Akt) is inhibited more substantially by the combination of Ptx and “TPE-Py-FFGYSA + light irradiation” (Ptx +; adjuvant +; light +), when compared with Ptx alone (Fig. 5A). Since p-Akt is a very important survival signal in cancer cells, earlier studies have demonstrated that constitutive expression of p-Akt undermines the sensitivity of cancer cells toward Ptx.^25^ It is thus proved in the present study that inhibition of the phosphorylation of Akt proteins plays a pivotal role in the sensitization effect of “TPE-Py-FFGYSA + light irradiation” on Ptx.

Furthermore, the down-stream apoptotic pathway was also evaluated using western blot. It is obvious that the combination of Ptx and “TPE-Py-FFGYSA + light irradiation” is much more effective in inducing mitochondria-originated apoptosis by increasing the cytoplasm expression of cytochrome-c (Cyt. c),^26^ compared with the other four control treatments. Moreover, the expression of one of the most important apoptotic markers, pro-caspase-3, undergoes the most attenuated signal under combinational treatment (Fig. 5A). What's more, the presence of *N*-acetylcysteine (NAC) as an antioxidant is able to significantly abolish the synergistic antitumor efficacy (Fig. 5B). Therefore, these results together elucidate the underlying synergistic mechanism, that is, the elevated intracellular ROS level resulting from “TPE-Py-FFGYSA + light irradiation” amplifies the action of Ptx by enhancing the inhibition of p-Akt, thus inducing mitochondria-originated apoptosis more efficiently (Fig. 5C).

## Conclusions

In summary, we have reported the synthesis and feasibility of TPE-Py-FFGYSA as an AIE adjuvant that has the combined capabilities of targeted imaging of EphA2 overexpressed in cancer cells and adjuvant amplification of antitumor efficacy of Ptx. TPE-Py-FFGYSA is weakly fluorescent in aqueous solution, and can selectively target EphA2 in PC-3 cancer cells along with its fluorescence turn on. By virtue of the simple incorporation of FFG as a self-assembly-aided unit, TPE-Py-FFGYSA can image EphA2 clusters in PC-3 cancer cells with a high contrast. Additionally, benefiting from the intracellular oxidative environment provided by TPE-Py-FFGYSA upon exposure to light, the antitumor activity of Ptx is significantly amplified with a synergistic effect of “0 + 1 > 1”. The combination treatment of Ptx with “TPE-Py-FFGYSA + light irradiation” gives an IC_50_ value as low as 7.8 nM, which is only 10.3% of that of Ptx alone. Western blot studies further reveal that such synergistic antitumor efficacy is rooted in the enhanced inhibition of p-Akt, which leads to a more effective inducement of mitochondria-originated apoptosis. This work thus extends the application scope of photosensitizers.
