# Supplementary material for: AIEgen-based theranostic system: targeted imaging of cancer cells and adjuvant amplification of antitumor efficacy of paclitaxel
Source: Chem Sci. 2016 Dec 2;8(3):2191–8. doi: 10.1039/c6sc03859j (PMC5407270; doi:10.1039/c6sc03859j)
Supplement: Supplementary file 1 [file SC-008-C6SC03859J-s001.pdf]

Electronic Supplementary Material (ESI) for Chemical Science.
This journal is © The Royal Society of Chemistry 2016

Electronic Supplementary Information

AIEgen-based theranostic system: targeted imaging of cancer cells and adjuvant amplification of antitumor efficacy of paclitaxel

Chao Chen,‡[a] Zhegang Song,‡[b] Xiaoyan Zheng,[b] Zikai He,[b] Bin Liu,*[c] Xuhui Huang,[b] Deling Kong,[a] Dan Ding*[a] and Ben Zhong Tang*[b]

Figures

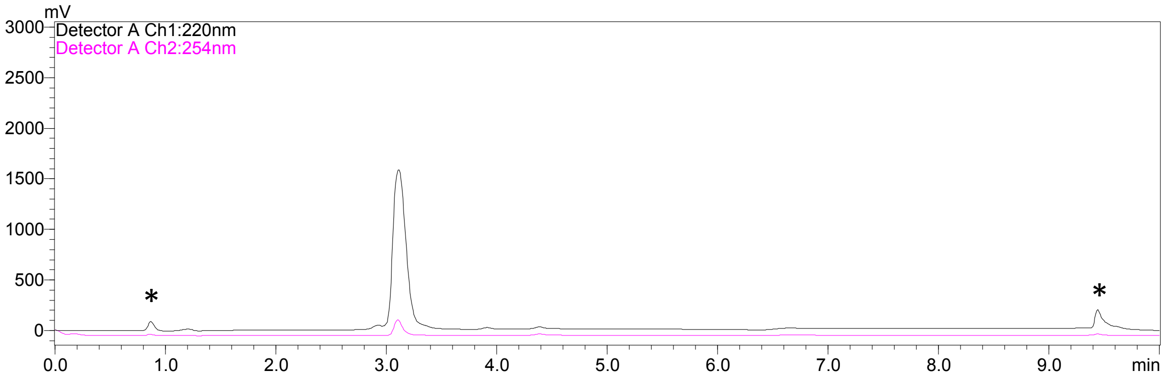

Fig. S1 LC spectrum of NH_2-FFGYSA (the stars represent systemic peaks).

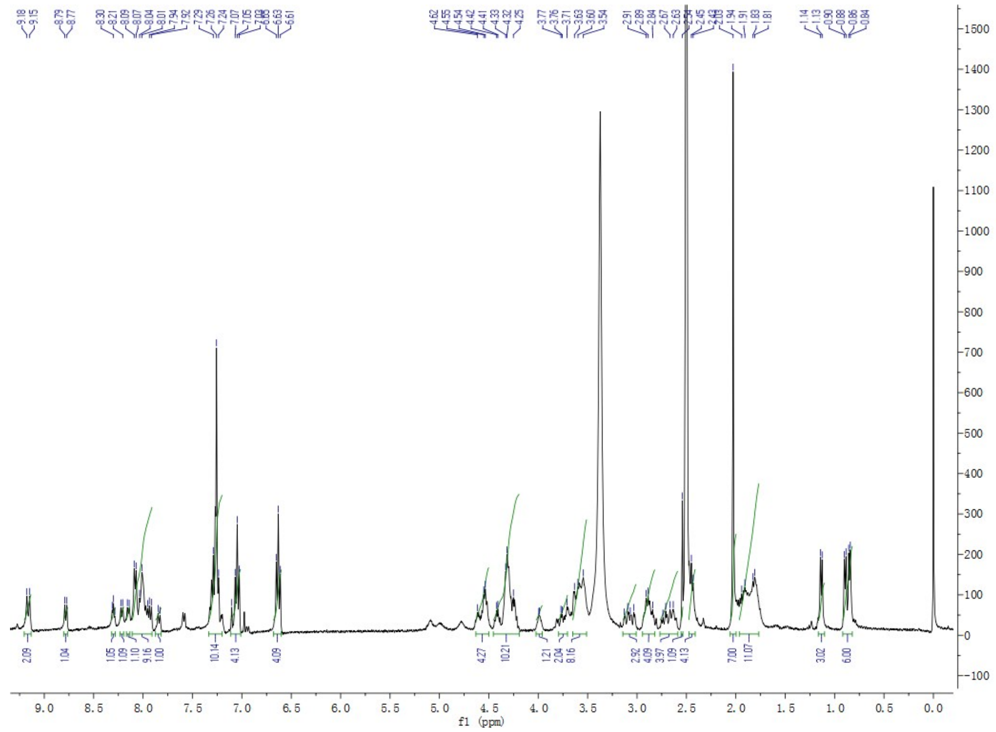

Fig. S2 ^{1}H NMR spectrum of NH_2-FFGYSA in DMSO-d_6.

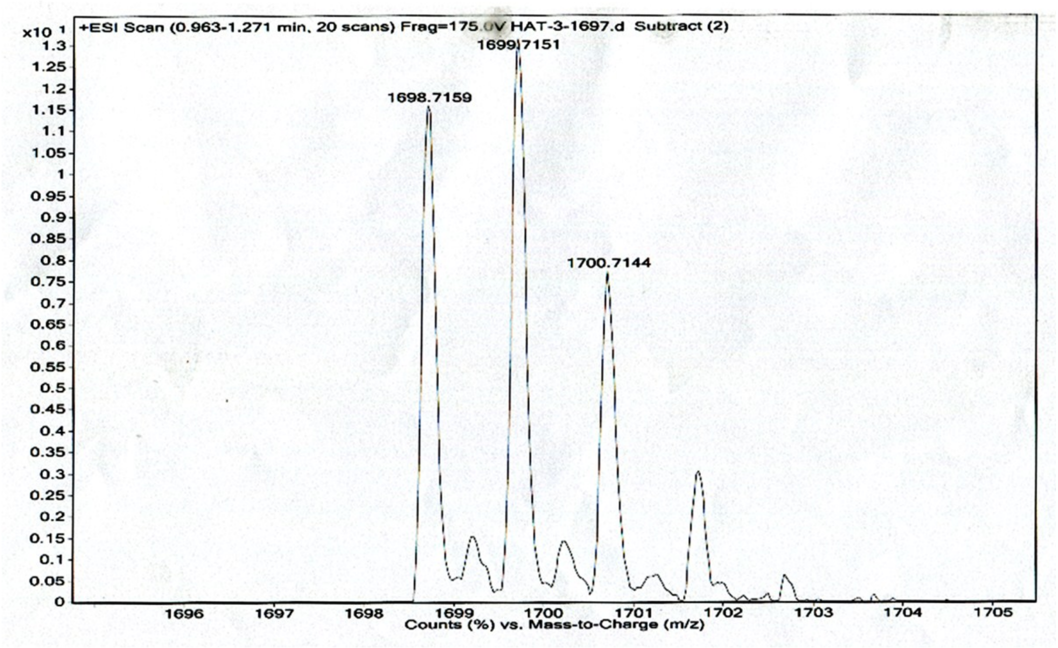

Fig. S3 HRMS spectrum of NH_2-FFGYSA.

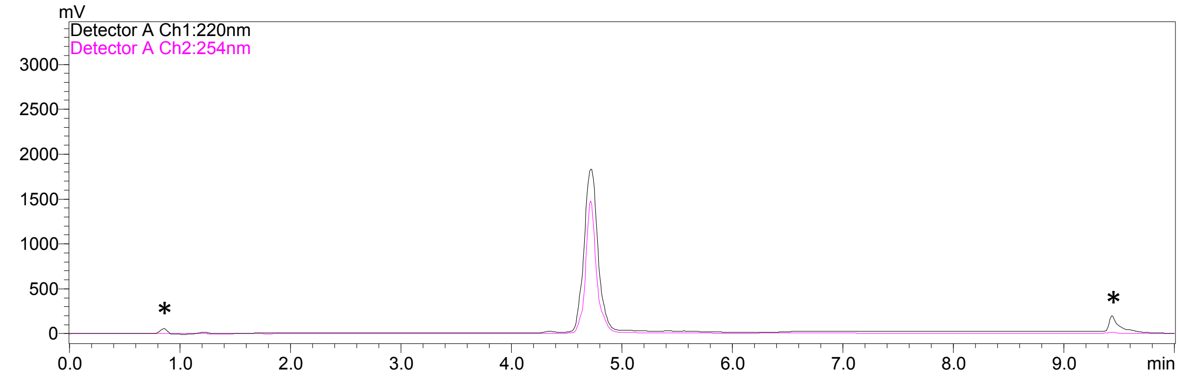

Fig. S4 LC spectrum of TPE-Py-FFGYSA (the stars represent systemic peaks).

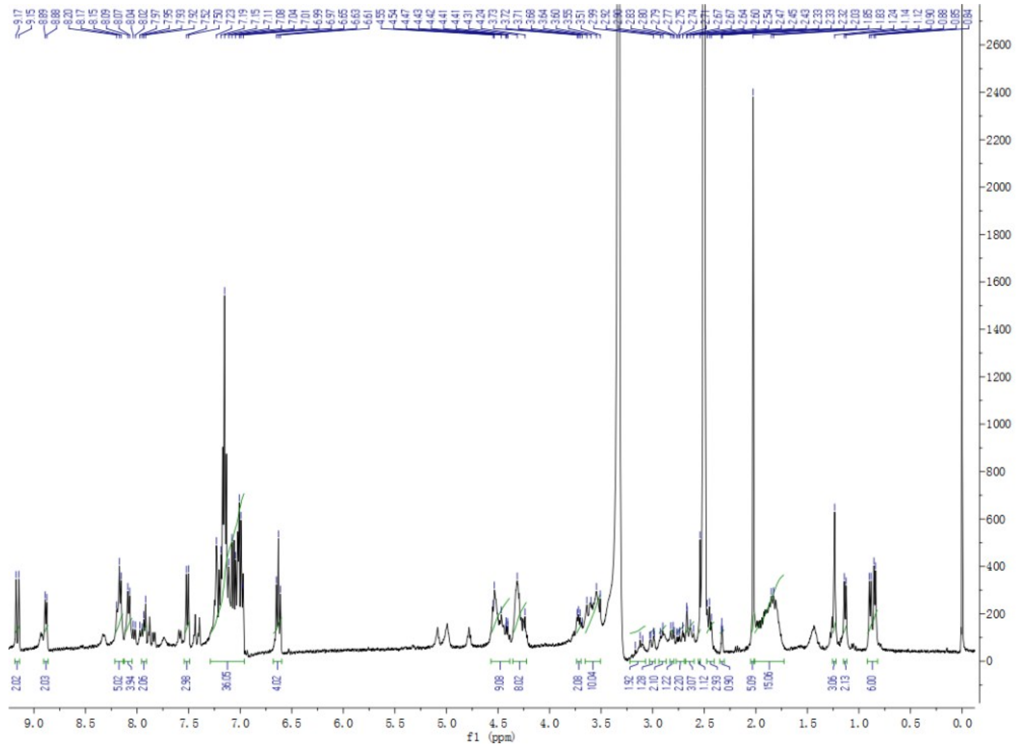

Fig. S5 ^{1}H NMR spectrum of TPE-Py-FFGYSA in DMSO-d_6.

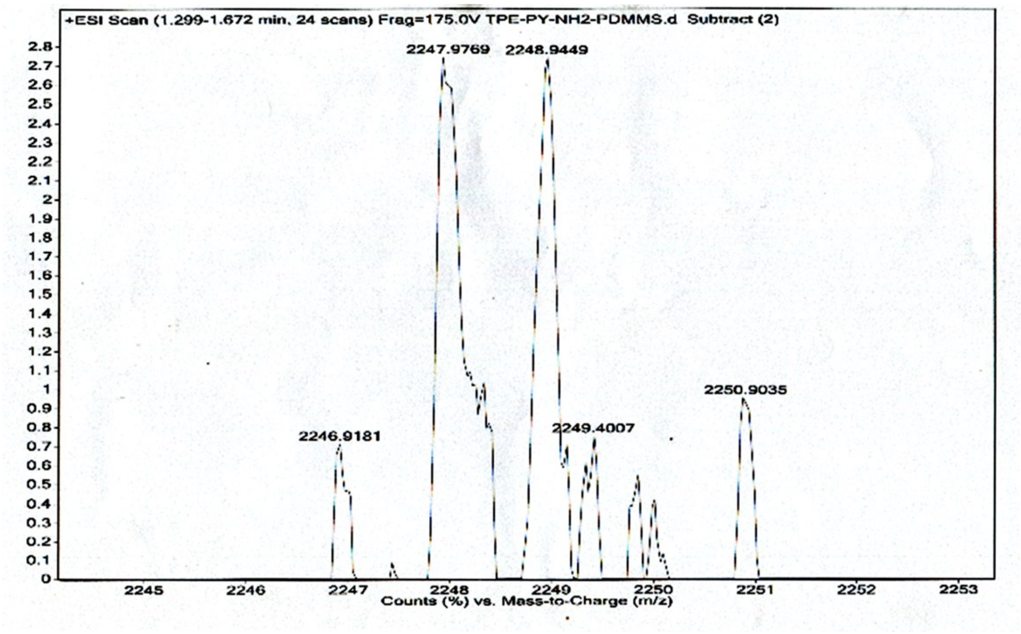

Fig. S6 HRMS spectrum of TPE-Py-FFGYSA.

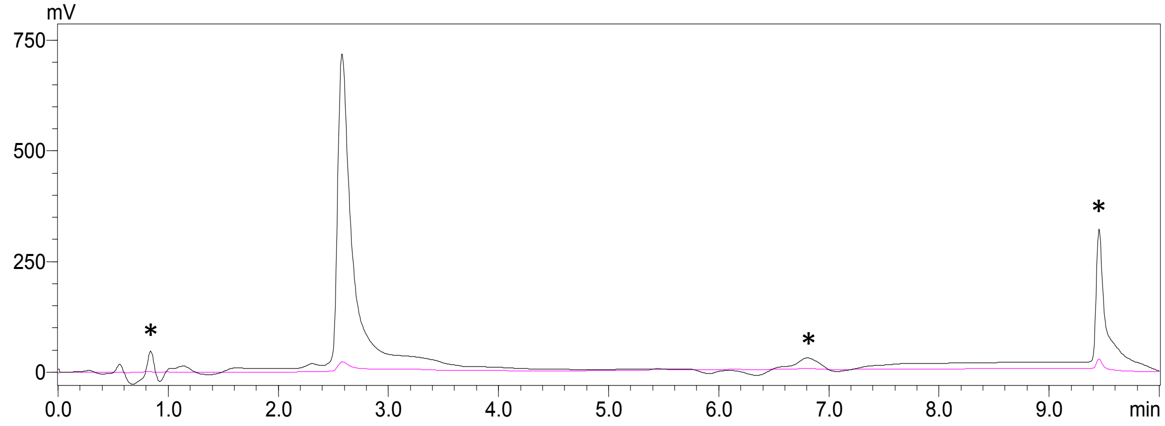

Fig. S7 LC spectrum of NH_2-YSA (the stars represent systemic peaks).

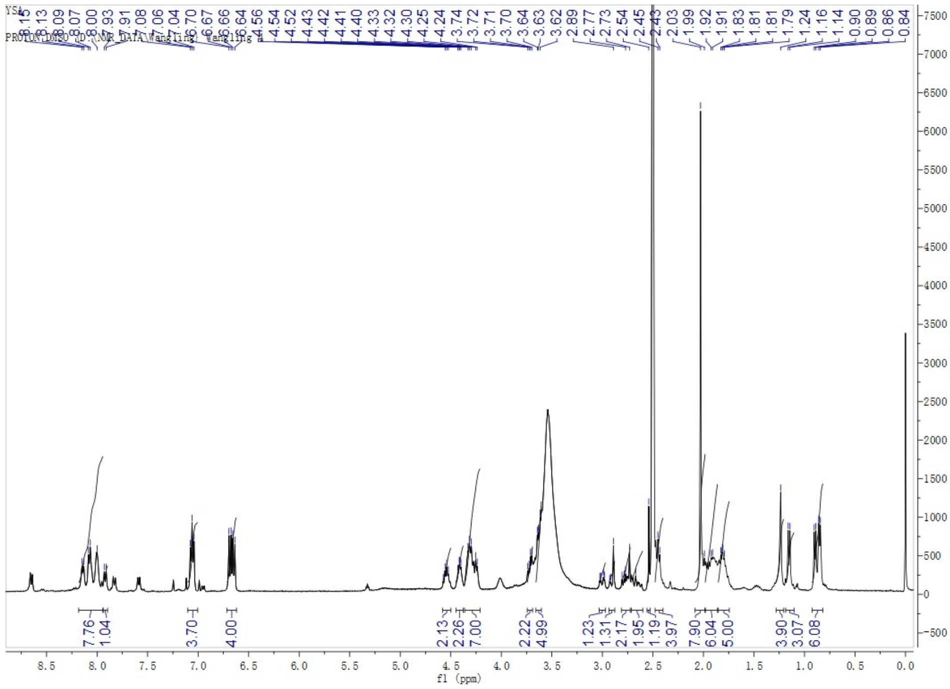

Fig. S8 ^{1}H NMR spectrum of NH_2-YSA in DMSO-d_6.

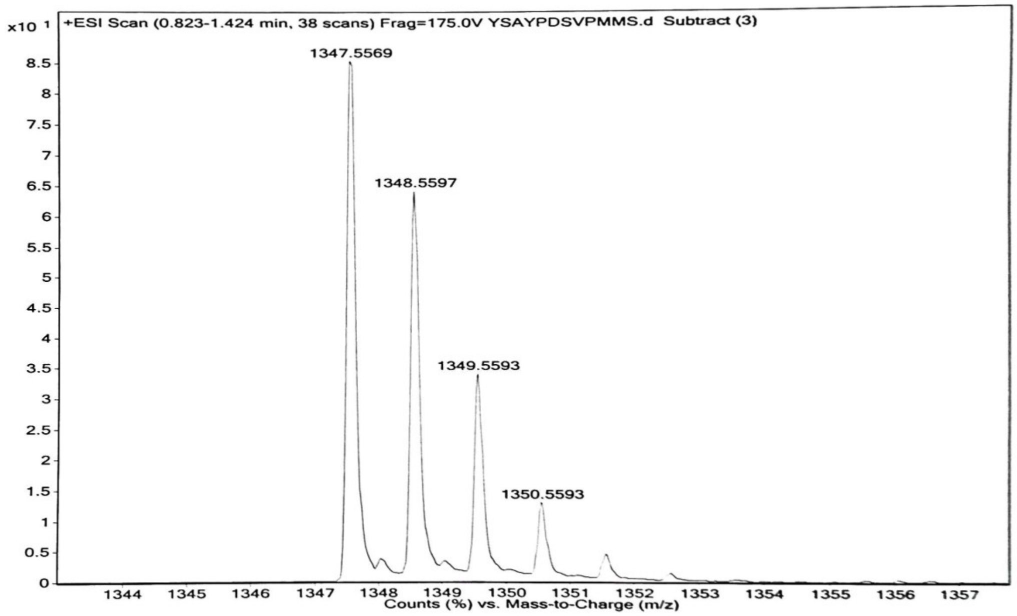

Fig. S9 HRMS spectrum of NH_2-YSA.

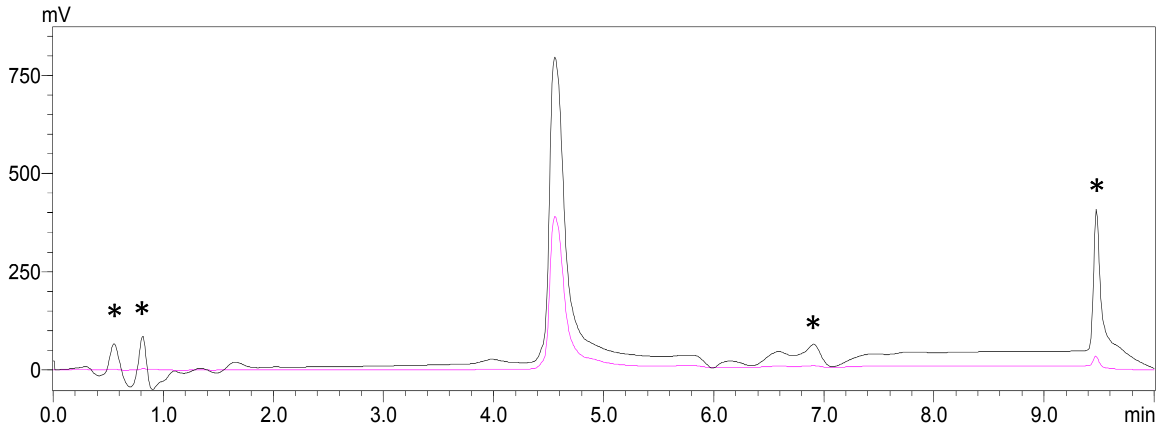

Fig. S10 LC spectrum of TPE-Py-YSA (the stars represent systemic peaks).

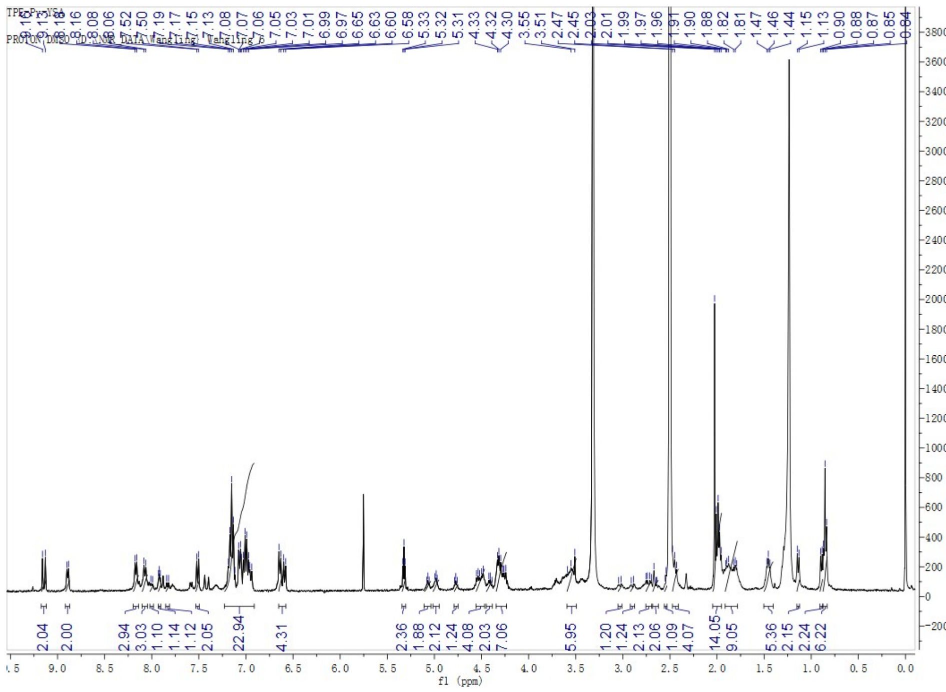

Fig. S11 ^{1}H NMR spectrum of TPE-Py-YSA in DMSO-d_6.

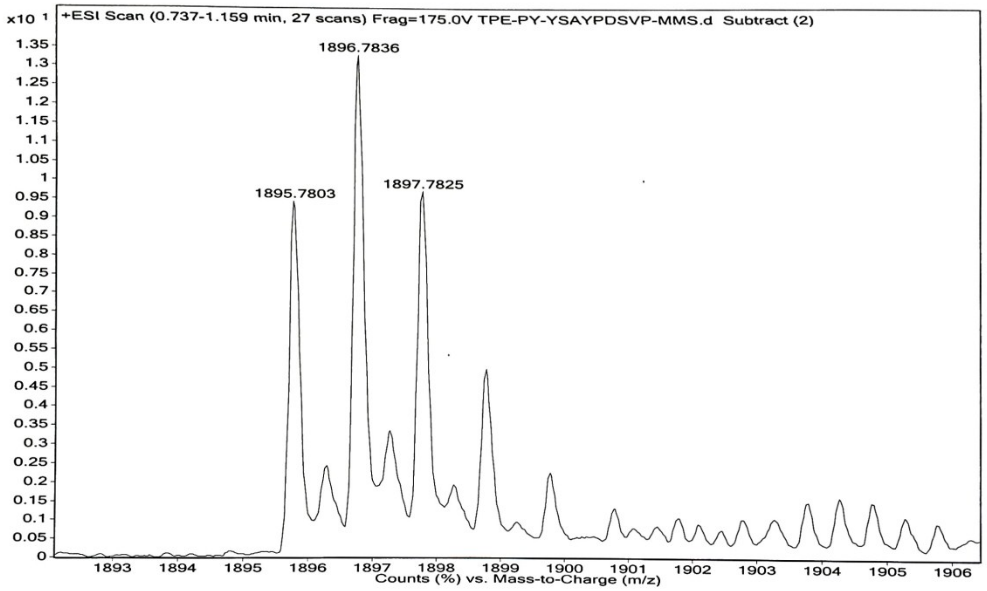

Fig. S12 HRMS spectrum of TPE-Py-YSA.

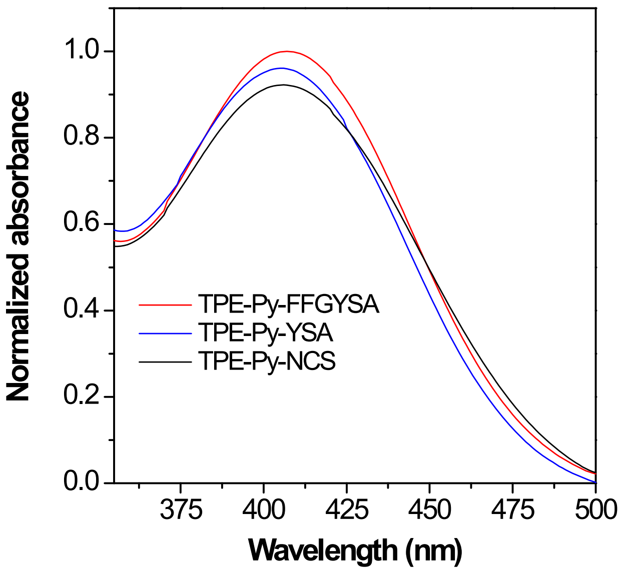

Fig. S13 Normalized UV-vis absorption spectra of TPE-Py-FFGYSA, TPE-Py-YSA, and TPE-Py-NCS in PBS buffer.

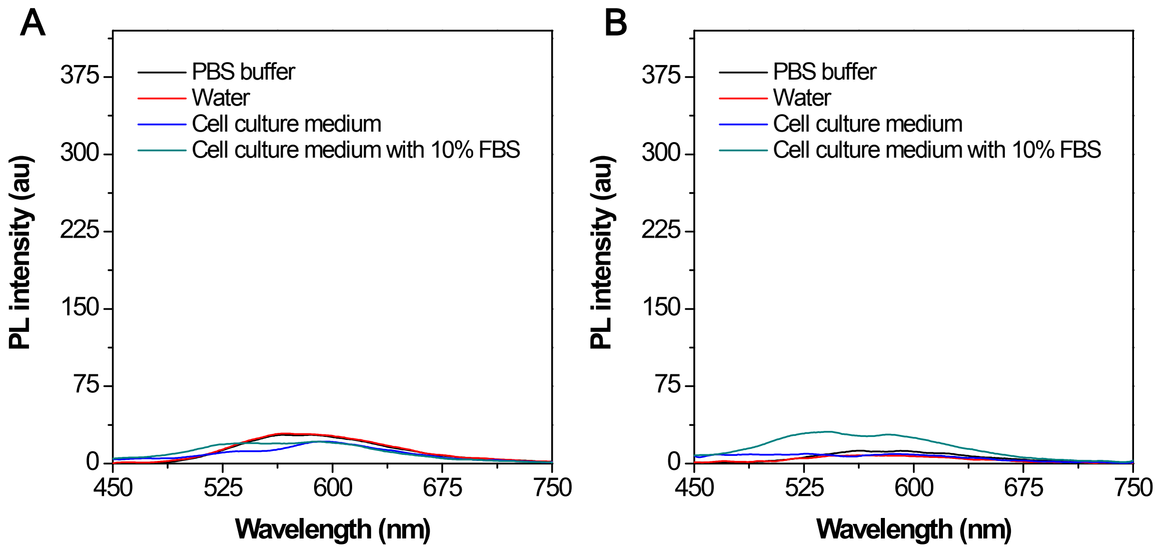

Fig. S14 PL spectra of (A) TPE-Py-FFGYSA and (B) TPE-Py-YSA in different solutions.

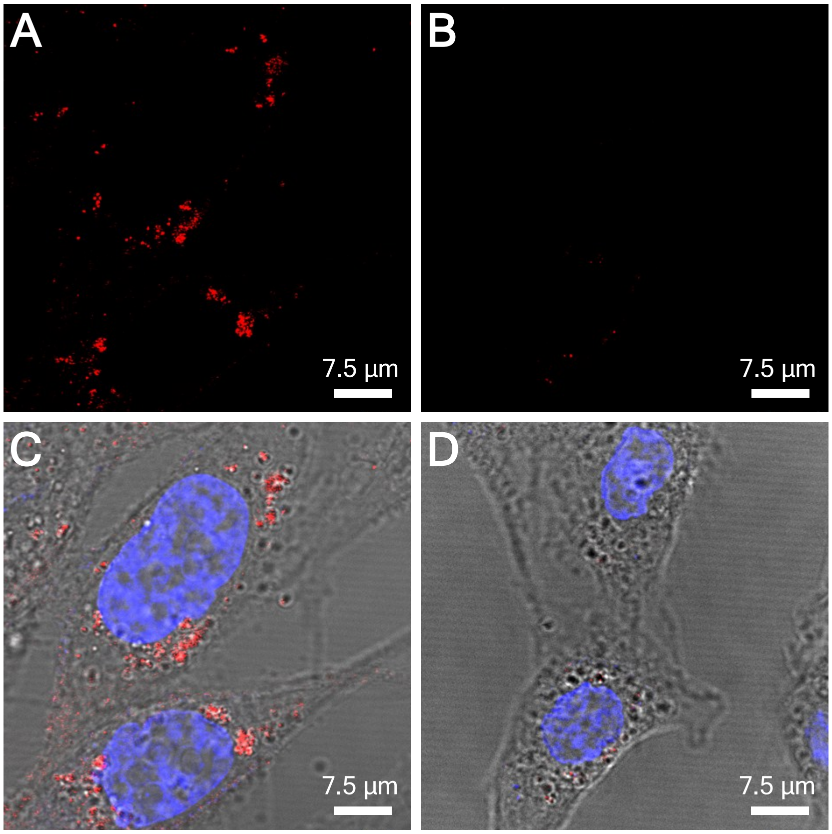

Fig. S15 CLSM images of (A) PC-3 cancer cells and (B) smooth muscle cells after staining with monoclonal anti-EphA2 antibody/Alexa Fluor 633-conjugated secondary antibody. (C) and (D) are the corresponding fluorescence/transmission overlay images of (A) and (B), respectively.

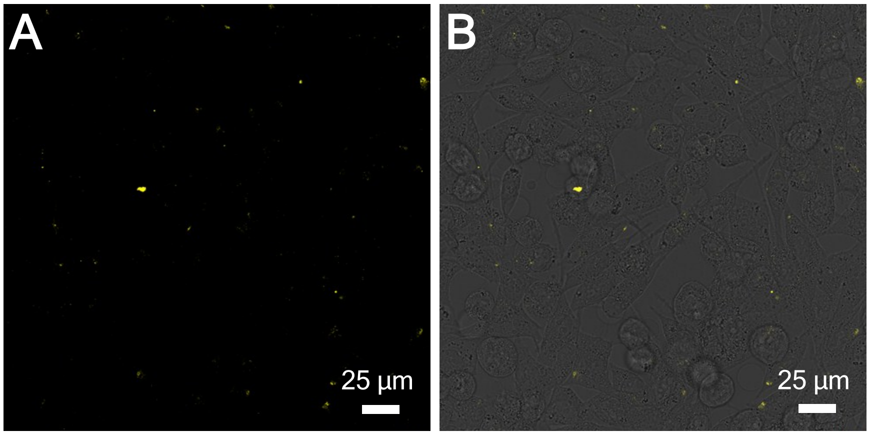

Fig. S16 (A) CLSM image of free YSA peptides (500 µM) pre-treated PC-3 cancer cells after incubation with TPE-Py-FFGYSA (1 µM) at 37 °C for 90 min. (B) is the corresponding fluorescence/transmission overlay image of (A).

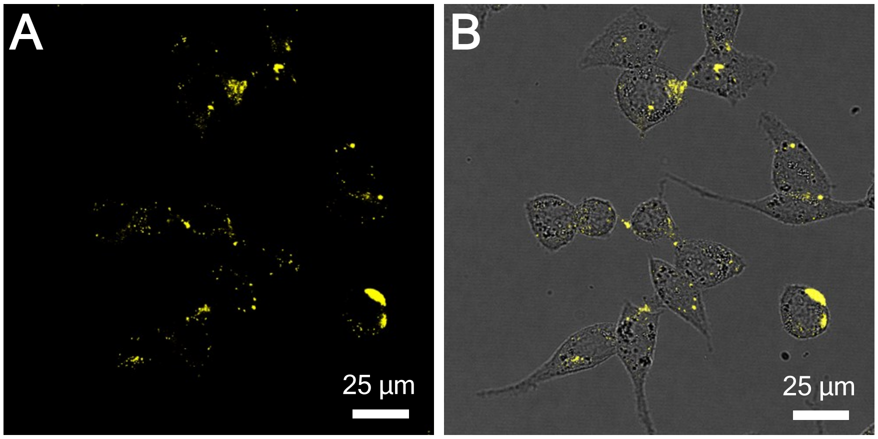

Fig. S17 (A) CLSM image of PC-3 cancer cells after incubation with TPE-Py-FFGYSA (1 µM) at 0 °C for 1 h, followed by further incubation of the cells at 37 °C for another 1 h. (B) is the corresponding fluorescence/transmission overlay image of (A).

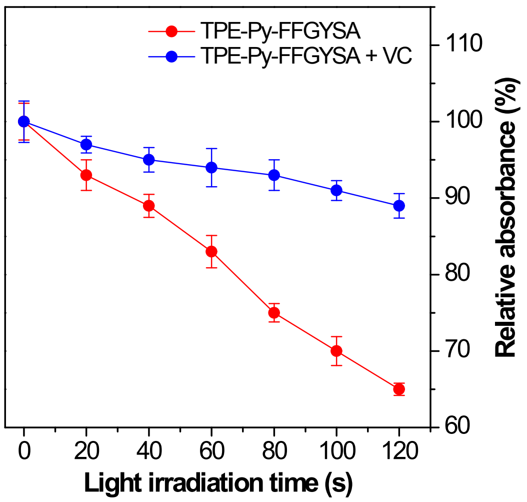

Fig. S18 Relative absorbance of 1,3-diphenylisobenzofuran (DPBF) at 418 nm as a function of light irradiation time of TPE-Py-FFGYSA (1 μM) in aqueous solution with and without addition of vitamin C (VC).

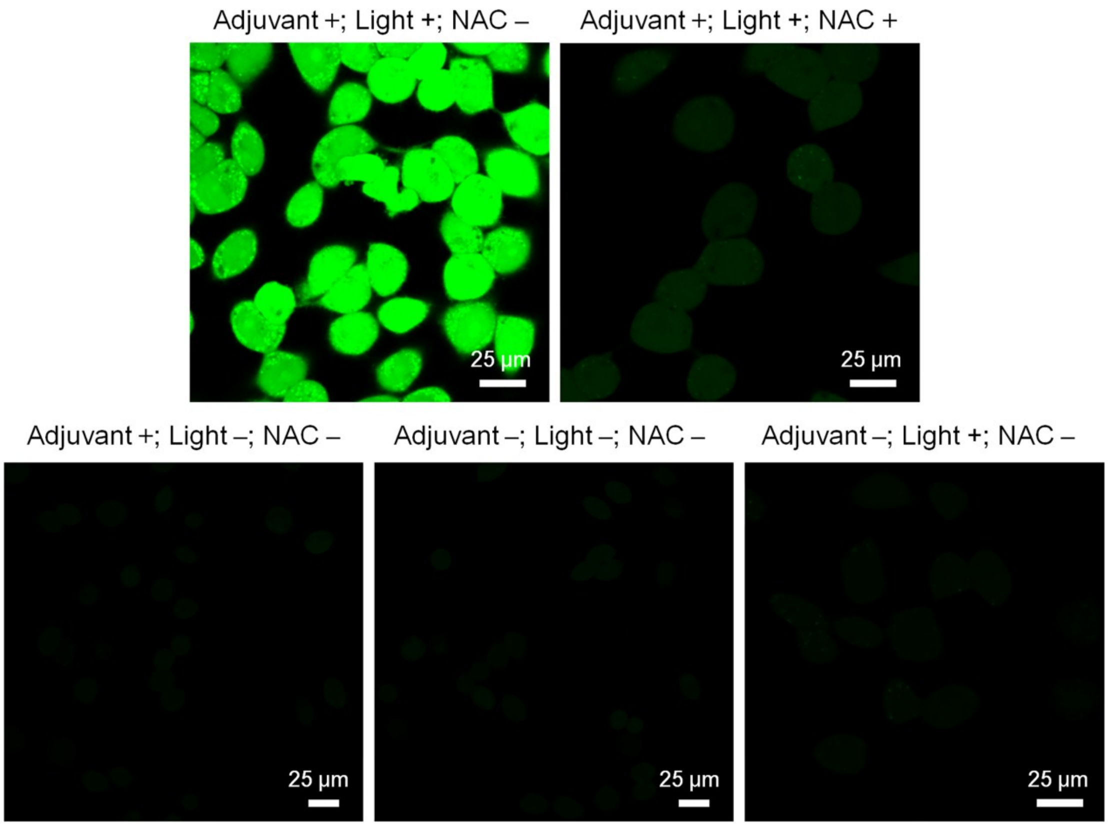

Fig. S19 CLSM images show the intracellular ROS levels of PC-3 cells received different treatments by using DCF-DA as the ROS indicator. The cells were incubated with TPE-Py-FFGYSA (1 μM) at 37 ^{o}C for 90 min. Light irradiation (0.1 W cm^{-2}) was performed for 2 min. [NAC] = 1 mM.

After incubation with TPE-Py-FFGYSA at 37 ^{o}C for 90 min, PC-3 cells were exposed to light irradiation for 2 min. Intense green emission from DCF can be distinctly seen inside the TPE-Py-FFGYSA-treated cells upon light irradiation (Adjuvant +; Light +; NAC –). Nevertheless, after the cells were pretreated with *N*-acetylcysteine (NAC), a ROS scavenger, the fluorescence intensity of DCF in the TPE-Py-FFGYSA-treated cells after light irradiation is greatly reduced (Adjuvant +; Light +; NAC +). The result indicates efficient ROS generation from TPE-Py-FFGYSA that targets EphA2 clusters in the PC-3 cancer cells. In comparison, the TPE-Py-FFGYSA-treated cells without exposure to light (Adjuvant +; Light –; NAC –) exhibit similarly week green fluorescence to the control cells (Adjuvant –; Light –; NAC – and Adjuvant –; Light +; NAC –), suggesting that light irradiation is necessary for TPE-Py-FFGYSA to produce ROS.

Experimental section

1. Chemicals

Fmoc-OSu and other Fmoc-amino acids were purchased from GL Biochem (Shanghai, China). 2-Cl-trityl chloride resin (1.0-1.2 mmol/g) was bought from Nankai University Resin Co. Ltd. Recombinant Human EphA2 was purchased from R&D Systems, Bio-Techne Co. Ltd. Monoclonal anti-EphA2 antibody, anti-phosphate-Akt, anti-Pro-Caspase-3, anti-Bcl-2 and anti-Cytochrome-c were obtained from Abcam. Alexa Fluor 633-conjugated second antibody was purchased from Life Technologies. Tetrahydrofuran (THF) and dichloromethane (DCM) were distilled from sodium benzophenone ketyl or calcium hydride, respectively, under nitrogen immediately prior to use. Other chemicals were obtained from Sigma-Aldrich, which were used as received without further purification.

2. Characterization

^{1}H and ^{13}C NMR spectra were measured on a Bruker ARX 400 NMR spectrometer using DMSO-d_6 as a solvent and tetramethylsilane (TMS; $\delta = 0$) was chosen as an internal reference. UV-vis absorption and PL spectra were recorded on Shimadzu UV-1700 spectrometer and Perkin-Elmer LS 55 spectrofluorometer, respectively. HPLC was carried out at a LUMTECH HPLC (Germany) system using a C_{18} RP column with MeOH (0.1% of TFA) and water (0.1% of TFA) as the eluents. LC-MS was conducted at the LCMS-20AD (Shimadzu) system. Cell imaging studies were performed by confocal laser scanning microscopy (CLSM, Zeiss LSM 410, Jena, Germany).

3. Synthesis and characterization of TPE-Py-NCS

TPE-Py-NCS was synthesized according to our previous report.[1] ^{1}H NMR (400 MHz, DMSO-d_6), δ (ppm): 8.87 (d, J = 6.4 Hz, 2H), 8.17 (d, J = 6.4 Hz, 2H), 7.88 (d, J = 16.4 Hz, 1H), 7.51 (d, J = 8.0 Hz, 2H), 7.41 (d, J = 16.4 Hz, 1H), 7.14–7.12 (m, 9H), 7.03 (d, J = 12.8 Hz, 2H), 7.01–6.96 (m, 6H), 4.49 (t, 2H), 3.72 (t, 2H), 1.96 (m, 2H), 1.65 (m, 2H). ^{13}C NMR (100 MHz, DMSO-d_6), δ (ppm): 151.9, 145.2, 143.6, 142.8, 142.5, 142.3, 141.4, 140.1, 139.8, 132.8, 131.0, 130.4, 130.2, 127.8, 127.6, 127.4, 126.7, 126.5, 123.5, 123.1, 58.8, 44.1, 27.7, 25.7. HRMS (MALDI-TOF), *m/z* calcd. for $C_{38}H_{33}N_2S^+$: 549.2359; found 549.2316 (M^+).

4. Synthesis of NH_2-FFGYSA

The peptide of NH_2-FFGYSA was synthesized by standard Fmoc solid-phase peptide synthesis (SPPS) using 2-chlorotrityl chloride resin and the corresponding N-Fmoc protected amino acids with side chains properly protected. The growth of the peptide chain was according to the established Fmoc SPPS protocol. After the last coupling step, excessive reagents were removed by a single *N,N*'-dimethylformamide (DMF) wash for 5 min (5 mL per gram of resin), followed by 5 steps of washing with DCM for 1 min (5 mL per gram of resin). The peptide derivative was cleaved using 95% of trifluoroacetic acid (TFA) with 2.5% of trimethylsilane (TMS) and 2.5% of H_2O for 30 min. 20 mL per gram of resin of ice-cold diethylether was then added to the cleavage reagent. Afterward, the obtained product was purified by HPLC. The peptide was obtained in 76% yield. ^{1}H NMR (400 MHz, DMSO-d_6), δ (ppm): 9.13–9.20 (d, J = 11.15 Hz, 2H), 8.76–8.81 (d, J = 8.12Hz, 1H), 8.28–8.32 (m, 1H), 8.19–8.23 (m, 1H), 8.13–8.17 (m, 1H), 7.91–8.11 (m, 9H), 7.82–7.87 (m, 1H), 7.20–7.33 (m, 10H), 7.01–7.11 (m, 4H), 6.60–6.68 (t, 4H), 4.50–4.63 (m, 4H), 4.19–4.45 (m, 10H), 3.96–4.02 (m, 1H), 3.70–3.79 (m, 2H), 3.51–3.65 (m, 8H), 3.01–3.14 (m, 3H), 2.82–2.94 (m, 4H), 2.59–2.77 (m, 4H), 2.54 (s, 1H), 2.41–2.47 (m, 4H), 2.03 (s, 7H), 1.77–1.96 (m, 11H), 1.10–1.16 (d, J = 7.07 Hz, 3H),

0.82–0.92 (m, 6H). HRMS, *m/z* calcd. for $C_{79}H_{107}N_{15}O_{23}S_2^+$: 1697.7106; found 1698.7159 (M + H)$^+$.

5. Synthesis of TPE-Py-FFGYSA

To prepare TPE-Py-FFGYSA, the peptide of NH_2-FFGYSA (14.7 mg, 8.7 μmol) was added to a solution of TPE-Py-NCS (2 mg, 2.9 μmol) in dimethyl sulfoxide (DMSO). *N,N*-Diisopropylethylamine (DIPEA) was used to adjust the final pH of the mixture to 8-9. After reaction for 24 h at room temperature, the final product was purified by HPLC to yield TPE-Py-FFGYSA in 70% yield. ^{1}H NMR (400 MHz, DMSO-d_6), δ (ppm): 9.14–9.19 (d, J = 10.97 Hz, 2H), 8.86–8.91 (d, J = 6.39 Hz, 2H), 8.14–8.22 (m, 5H), 8.05–8.12 (m, 4H), 7.91–7.96 (m, 2H), 7.49–7.54 (d, J = 8.25 Hz, 3H), 6.96–7.29 (m, 36H), 6.59–6.68 (t, 4H), 4.39–4.57 (m, 9H), 4.22–4.36 (m, 8H), 3.70–3.74 (m, 2H), 3.50–3.65 (m, 10H), 3.07–3.22 (m, 2H), 2.97–3.03 (m, 1H), 2.87–2.94 (m, 2H), 2.79–2.83 (m, 1H), 2.69–2.77 (m, 2H), 2.60–2.68 (m, 3H), 2.54 (s, 1H), 2.40–2.48 (m, 3H), 2.30–2.35 (m, 1H), 2.03 (s, 5H), 1.73–2.01 (m, 15H), 1.24 (s, 3H), 1.12–1.15 (d, J = 6.92 Hz, 2H) , 0.82–0.92 (m, 6H). HRMS, *m/z* calcd. for $C_{117}H_{140}N_{17}O_{23}S_3^+$: 2246.9465; found 2247.9769 (M + H)$^+$.

6. Synthesis of NH_2-YSA

The peptide of NH_2-YSA was synthesized according to the same procedures as that for NH_2-FFGYSA, which was obtained in 82% yield. ^{1}H NMR (400 MHz, DMSO-d_6), δ (ppm): 8.18–7.94 (m, 8H), 7.92 (d, J = 8.0 Hz, 1H), 7.10–7.00 (m, 4H), 6.67 (dd, J = 15.0, 8.4 Hz, 4H), 4.55 (dd, J = 13.4, 6.9 Hz, 2H), 4.41 (dd, J = 7.2, 5.0 Hz, 2H), 4.36–4.21 (m, 7H), 3.72 (dd, J = 11.0, 5.2 Hz, 2H), 3.66–3.60 (m, 5H), 3.00 (dd, J = 14.3, 4.8 Hz, 1H), 2.94–2.88 (m, 1H), 2.77 (dd, J = 17.6, 11.9 Hz, 2H), 2.67 (s, 2H), 2.54 (s, 1H), 2.44 (d, J = 7.4 Hz, 4H), 2.01 (d, J = 15.0 Hz, 8H), 1.92 (d, J = 5.5 Hz, 6H), 1.81 (dd, J = 8.1, 5.8 Hz, 5H), 1.24 (s, 4H), 1.15 (d, J =

7.1 Hz, 3H), 0.87 (dd, J = 17.8, 6.6 Hz, 6H). HRMS, *m/z* calcd. for $C_{59}H_{86}N_{12}O_{20}S_2^+$: 1346.5523; found 1347.5569 $(M + H)^+$.

7. Synthesis of TPE-Py-YSA

TPE-Py-YSA was synthesized according to the same procedures as that for TPE-Py-FFGYSA, which was obtained in 74% yield. ^1H NMR (400 MHz, DMSO-d_6), δ (ppm): 9.14 (d, J = 12.2 Hz, 2H), 8.89 (d, J = 6.8 Hz, 2H), 8.17 (d, J = 6.8 Hz, 3H), 8.07 (d, J = 8.0 Hz, 3H), 8.00 (d, J = 7.8 Hz, 1H), 7.93–7.91 (m, 1H), 7.83 (d, J = 8.2 Hz, 1H), 7.51 (d, J = 8.3 Hz, 2H), 7.23–6.91 (m, 23H), 6.61 (dd, J = 20.4, 8.3 Hz, 4H), 5.32 (t, J = 4.7 Hz, 2H), 5.11–5.04 (m, 2H), 5.02–4.95 (m, 2H), 4.79–4.75 (m, 1H), 4.56–4.47 (m, 4H), 4.45–4.38 (m, 2H), 4.34–4.23 (m, 7H), 3.59–3.49 (m, 6H), 3.03 (d, J = 10.4 Hz, 1H), 2.90 (d, J = 9.3 Hz, 1H), 2.73 (dd, J = 16.6, 6.2 Hz, 2H), 2.68–2.62 (m, 2H), 2.55 (d, J = 3.9 Hz, 1H), 2.47–2.41 (m, 4H), 2.05–1.95 (m, 14H), 1.91–1.78 (m, 9H), 1.50–1.40 (m, 5H), 1.14 (d, J = 7.1 Hz, 2H), 0.89 (d, J = 6.7 Hz, 2H), 0.85 (t, J = 6.6 Hz, 6H). HRMS, *m/z* calcd. for $C_{97}H_{119}N_{14}O_{20}S_3^+$: 1895.7882; found 1895.7803 (M^+).

8. Cell culture

PC-3 cancer cells and smooth muscle cells were cultured in Dulbecco's Modified Eagle’s Medium (DMEM) containing 10% fetal bovine serum (FBS) and 1% penicillin-streptomycin at 37 ^oC in a humidified environment containing 5% CO_2, respectively. Before experiments, the cells were pre-cultured until confluence was reached.

9. EphA2 imaging in cells

PC-3 cancer cells or human smooth muscle cells were seeded in confocal imaging chambers at a density of 1×10^5 cells. After 12 h, the cells were incubated with TPE-Py-FFGYSA or TPE-

Py-YSA at the concentration of 1 μM in serum-free cell culture medium at 37 ^{o}C for 90 min. Subsequently, the live cells were washed three times with 1 × PBS buffer, which was followed by imaging with CLSM upon excitation at 405 nm and a collection of fluorescent signal at 580 ± 20 nm. To investigate the feasibility of TPE-Py-FFGYSA in tracing the intracellular movement of EphA2, PC-3 cancer cells in confocal imaging chambers were placed on ice for 30 min, followed by incubation with 1 μM of TPE-Py-FFGYSA in pre-cooled cell culture medium on ice for 60 min. After that, the cells were washed with 1 × PBS, fixed with 4% paraformaldehyde for 20 min on ice, and immediately imaged by CLSM. Alternatively, after incubation with 1 μM of TPE-Py-FFGYSA at 0 ^{o}C for 60 min, PC-3 cells were washed with 1 × PBS and incubated in fresh culture medium for another 10 and 60 min, respectively, followed by imaging with CLSM.

10. Staining with commercial antibodies

PC-3 cancer cells were incubated with 1 μM of TPE-Py-FFGYSA in serum-free cell culture medium at 37 ^{o}C for 90 min. After fixation with 4% paraformaldehyde, the PC-3 cells were incubated with 1% monoclonal anti-EphA2 antibody in 1 × PBS buffer for 2 h at room temperature. The cells were then washed three times with 1 × PBS and further incubated with Alexa Fluor 633-conjugated second antibody (10 μg mL^{-1}) for 90 min. Subsequently, the cells were washed three times with 1 × PBS and imaged by CLSM. For TPE-Py-FFGYSA: excitation at 405 nm and signal collection at 580 ± 20 nm. For Alexa Fluor 633: excitation at 633 nm and signal collection from 650 to 700 nm. Alternatively, the PC-3 cancer cells and smooth muscle cells without treatment with TPE-Py-FFGYSA were stained with anti-EphA2 antibody/Alexa Fluor 633-conjugated second antibody, which were then imaged with CLSM to evaluate the EphA2 levels in these two cell lines.

11. Intracellular detection of ROS

The ROS production of TPE-Py-FFGYSA in PC-3 cancer cells upon exposure to light was assessed using DCF-DA as the indicator. After incubation with 1 μM of TPE-Py-FFGYSA at 37 ^{o}C for 90 min in the dark, PC-3 cancer cells were incubated with 1 μM of DCF-DA for 5 min. Subsequently, the cells were washed with 1 × PBS and exposed to white light irradiation (0.1 W cm^{-2}) for 2 min, followed by imaging with CLSM. For DCF detection: excitation at 488 nm and signal collection at 530 ± 20 nm. Alternatively, before treatment with TPE-Py-FFGYSA, the cells were pre-treated with NAC (1 mM) for 2 h. In addition, the TPE-Py-FFGYSA-treated cells without light irradiation, the cells with only pure light irradiation, and the cells without any treatments were used as controls.

12. Theoretical calculations

To obtain the geometric and electronic structures of TPE-Py-Me, we performed the first principle calculations by Gaussian 09 program. The geometric structure of TPE-Py at the ground state (S_0) and the excited state (S_1) were optimized by density functional theory (DFT) and time-dependent density functional theory (TD-DFT) calculations at the B3LYP/6-31G* level, respectively. The transition energies for the most relevant singlet and triplet excited states for TPE-Py are further evaluated by CAM-B3LYP at the B3LYP/6-31G* level, respectively.

13. Study on synergistic antitumor efficacy

The adjuvant amplification of antitumor efficacy of Ptx by "TPE-Py-FFGYSA + light irradiation" was studied by MTT assays. PC-3 cancer cells were incubated with 1 μM of TPE-Py-FFGYSA at 37 ^{o}C for 90 min, which were then washed with 1 × PBS and exposed to 32 nM

of Ptx. Subsequently, single irradiation with white light (0.1 W cm^{-2}, 2 min) were carried out at 0, 3, 6, 9, or 12 h post addition of Ptx, which was followed by standard MTT test at 24 h. In brief, at 24 h post Ptx addition, the cells in 96-well plate were washed with 1 × PBS and 100 μL of freshly prepared MTT solution in culture medium (0.5 mg mL^{-1}) was added to each well. After 4 h incubation, the MTT solution was removed cautiously and then 100 μL of DMSO was added into each well. The plate was shaken for 10 min and then the absorbance of MTT at 490 nm was determined by the microplate reader (Genios Tecan). Furthermore, after incubation with 1 μM of TPE-Py-FFGYSA at 37 ^{o}C for 90 min, PC-3 cells were exposed to a series of doses of Ptx (including 0 nM). Irradiations with white light (0.1 W cm^{-2}, 2 min) were subsequently performed three times at 12, 24, and 36 h post Ptx addition, respectively. The MTT assays were conducted at 48 h post Ptx addition. In addition, the Ptx-treated cells received other "Adjuvant/Light" treatments including "Adjuvant +; Light –", "Adjuvant –; Light + (three times at 12, 24, and 36 h post addition of Ptx, respectively)" and "Adjuvant –; Light –" were used as controls.

14. Western blot analysis

Protein levels of p-Akt, Akt Cytochrome-c, and Pro-Caspase-3 in PC-3 cancer cells received various treatments (Ptx concentration was fixed at 8 nM) were analyzed by Western blot. In brief, at 48 h post Ptx addition, the PC-3 cell lysates were prepared, electrotransferred, and then immunoblotted with anti-phosphate-Akt, anti-Akt, anti-Cytochrome-c, and anti-Pro-Caspase-3. Detection was carried out with immobilion western chemiluminescent HRP substrate and imaging was conducted by a chemiluminescence imaging system (Clinx Science Instruments).

15. Statistical analysis

Quantitative data were expressed as mean ± standard deviation (SD). Statistical comparisons were made by ANOVA analysis and Student's *t*-test. *P* value < 0.05 is considered statistically significant.

References

1 W. Zhang, R. T. K. Kwok, Y. Chen, S. Chen, E. Zhao, C. Y. Y. Yu, J. W. Y. Lam, Q. Zheng and B. Z. Tang, *Chem. Commun.*, 2015, **51**, 9022.
